# Cost-effectiveness of pre-emptive pharmacogenetic testing: An umbrella review

**DOI:** 10.1371/journal.pone.0338277

**Published:** 2026-06-16

**Authors:** Taichi Ochi, Manon G. den Uil, Greta Piazza, Geert W. J. Frederix, Eelko Hak, Vera H. M. Deneer, Talitha L. Feenstra

**Affiliations:** 1 Department of PharmacoTherapy, -Epidemiology & -Economics, Groningen Research Institute of Pharmacy, University of Groningen, Groningen, the Netherlands‌‌; 2 University of Groningen Library, University of Groningen, Groningen, the Netherlands; 3 Division Laboratories, Pharmacy and Biomedical Genetics, Clinical Pharmacy, University Medical Centre Utrecht, Utrecht, the Netherlands; 4 Julius Center for Health Sciences and Primary Care, University Medical Center Utrecht, Utrecht University, Utrecht, the Netherlands; 5 Division of Pharmacoepidemiology and Clinical Pharmacology, Utrecht Institute for Pharmaceutical Sciences, Utrecht University, Utrecht, the Netherlands; 6 Center for Nutrition, Prevention and Health Services Research, National Institute for Public Health and the Environment, Bilthoven, the Netherlands; Shaheed Rajaei Cardiovascular Medical and Research Center: Rajaie Cardiovascular Medical and Research Center, IRAN, ISLAMIC REPUBLIC OF

## Abstract

**Background:**

Consortia have been established and published pharmacogenetic-specific guidelines with pharmacotherapeutic recommendations. While these consortia and their guidance addressed important information gaps, implementation barriers, including determining the cost-effectiveness for further implementation in healthcare practice, remain unaddressed.

**Methods:**

The authors systematically searched PubMed, EMBASE, and Web of Science from inception to March 2023 for systematic reviews of pharmacoeconomic evaluations of PGx studies. A second search was conducted in October 2025. The studies from the databases were imported into Rayyan for screening the title and abstract, based on the inclusion and exclusion criteria. The umbrella review was conducted in adherence to the proposal registered in PROSPERO (CRD42023408535). The quality of eligible systematic reviews was assessed by the AMSTAR-2 checklist.

**Results:**

From the initial screening of the three databases, title and abstract screening, and full paper readings, 17 systematic reviews were included for the umbrella review. The evidence points to the fact that with an increasing number of evaluations, the evidence for the cost-effectiveness of PGx testing was increasing over the years. From the included reviews, data from 211 unique evaluation studies were extracted. 150 (71.1%) studies reported that PGx testing was cost-effective. The most frequently used type of economic evaluation was cost-utility analysis (n = 78). Most economic evaluations investigated the pharmacogene *CYP2C19* (n = 46), followed closely by *CYP2C9/VKORC1* (n = 28).

**Conclusions:**

The number of economic evaluations demonstrating cost-effectiveness of PGx testing has increased over the years. However, the majority of these evaluations were conducted in a small number of countries. When interpreting findings for different countries, the evaluations need to be adjusted on a case-by-case basis for applicability.

## Introduction

Pharmacogenetic (PGx) tests are used to determine whether a patient carries genetic variants that influence the pharmacokinetics, effectiveness, or safety of drugs. Several consortia, such as the Dutch Pharmacogenetics Working Group (DPWG), Clinical Pharmacogenetics Implementation Consortium (CPIC), the Canadian Pharmacogenomics Network for Drug Safety (CPNDS), and the French National Network (Réseau) of Pharmacogenetics (RNPGx), were established and publish PGx-specific guidelines with pharmacotherapeutic recommendations [1]. Another working group, the Association for Molecular Pathology Pharmacogenetics working group (AMP PGx), provides recommendations on allele selection for PGx tests [2].

While these consortia and their guidance addressed important information gaps, such as pharmacotherapeutic recommendations for patients with specific genotypes (i.e., drug-gene interactions), other implementation barriers for further clinical implementation of PGx were left unaddressed [3]. Several studies demonstrate the efficacy of PGx or how to overcome practical barriers, e.g., selecting clinically relevant PGx tests (i.e., genetic variants to be tested), translation of test results into clinical guidance, and the need for clinical decision support has been addressed to fill this gap [4–6]. To further increase the understanding of PGx, several initiatives have focused on postgraduate education of pharmacists and physicians on using PGx in daily practice [4–6]. Thus, important steps have been taken to support the implementation of PGx. Yet, one persistent issue remains – the cost of PGx. Data on cost-effectiveness has been shown to be inconclusive, which influences the decisions on reimbursement of the PGx tests by health insurers [3]. Such cost-effectiveness analyses are beyond the scope of existing consortia guidelines [7].

The amount of evidence on the cost-effectiveness of individual PGx tests and panel-based approaches has been increasing. Incorporating these genetic tests requires careful consideration in light of rapid changes in the field [8–10]. Reimbursement by health insurers has been increasing over recent years in the United States [11]. This has been supported by the dramatic decrease in the cost of genetic tests over the last two decades. As the cost of PGx tests decreases, a re-evaluation of previous cost-effectiveness evaluations may shed light on whether the current pricing impacts these evaluations [12]. To compare economic evaluations of PGx, a synthesis of studies is required, as previous systematic reviews extracted different data. As some systematic reviews focused on single diseases, the applicability and scope of cost-effectiveness across PGx testing have been limited [13,14].

This study aims to provide an overview of economic evaluations of genetic tests to evaluate the cost-effectiveness of PGx. Rather than adding to the literature of systematic reviews, an umbrella review was conducted. An umbrella review includes only reviews with the highest level of evidence: systematic reviews and meta-analyses [15]. By conducting an umbrella review, the study will avoid replication of previous efforts and synthesise the findings of the included systematic reviews. Focusing on economic evaluations allows the study to include all genes, diseases, or medicines. From the data extracted, we aim to shed light on gaps in the literature pertaining to the cost-effectiveness of PGx. Thereby identifying the potential effects of cost-trends and outlining recommendations to facilitate policy decisions concerning the implementation of pre-treatment PGx testing.

## Methods

### Umbrella review methods

The authors systematically searched, organised, and evaluated data from systematic reviews and meta-analyses on pharmacoeconomic evaluations of PGx studies, taking care to avoid double-counting of overlapping studies. The umbrella review guideline by the JBI Manual for Evidence Synthesis was followed [16]. The umbrella review was conducted in adherence to a proposal that was registered in PROSPERO (CRD42023408535). In addition, the PRIMSA 2020 Checklist was followed and completed [17] (S1 Table).

### Literature search and screening

The authors systematically searched PubMed, EMBASE, and Web of Science from the inception to March 2023 for systematic reviews of pharmacoeconomic evaluations of PGx studies. A second search was conducted in October 2025 to address any new systematic reviews that had been published since the initial search. An abridged version of the search strategy is as follows (terms used for title and abstract search): (pharmacoeconomic OR cost-benefit OR cost-effectiveness OR cost-utility) AND (pharmacogenetic OR pharmacogenomic OR polymorphism, single nucleotide) AND (personalized medicine OR precision medicine). The full search terms can be found in S2 Table. After de-duplication of results from the various databases, the systematic reviews were ready for selection. Definitions of terms used can be found in Table 1. The studies from the databases were then imported into Rayyan for screening the title and abstract, based on the inclusion and exclusion criteria [18].

**Table 1:** Definitions of terms used in the paper.

Term	Definition
Genetic Biomarker	A DNA or RNA characteristic that is an indicator of response to therapeutic or other intervention.
Pharmacogenetics (PGx)	Genetic variants of pharmacogenes can affect individual responses to drugs, both in terms of therapeutic effect as well as side effects.
Pharmacogene	Genes in drug metabolic pathways whose variations affect drug response in an individual. Targets of pharmacogenetic testing.
Polygenic Risk Score	A value estimate of an individual’s common genetic liability to a phenotype, calculated as a sum of their genome-wide genotypes and weighted by corresponding genotype effect size estimates derived from summary statistic GWAS data.

GWAS: Genome-wide association study.

### Study selection and eligibility criteria

Two authors (TO and MU) screened the titles and abstracts independently. This was followed by full text review. Any discrepancies in selecting articles were first discussed between TO and MU. If no consensus was reached, the discrepancies were resolved by consulting a third investigator (TF or VD). The references cited in all eligible articles were also manually searched. If a review-study was not found within the search criteria but fit the eligibility criteria, it was added to the final list of eligible studies as part of snowball sampling.

Studies included were systematic reviews of economic evaluations of PGx studies, economic evaluations of drug-gene interactions based on guidelines, and economic evaluations of genetic studies on variants that impact drug response. Systematic reviews of genetic tests not related to PGx were excluded (i.e., tumour genetics, prognostic markers). Economic evaluation study types that were included were cost-effective analyses, cost-utility analyses, cost-benefit analyses, and cost-minimisation analyses. The selected full-text documents were read by TO and MU for final selection and data extraction.

### Data extraction

The retrieved data for the systematic reviews included: name of the first author, year of publication, category of studies included in the review (general or disease specific), geographical location, study objective, databases searched, number of included economic evaluation studies, years covered, quality assessment, main conclusions, and limitations. As the aim was to synthesise extracted data from selected systematic reviews, no further comparisons between the systematic reviews were conducted.

From the systematic reviews, data from the economic evaluations were extracted. The extracted data included: geographical location, drug, PGx test, genetic variant, disease variant, test cost, economic evaluation type, quantitative results, conclusion regarding cost-effectiveness, and alignment to PGx guidelines. For the full list and data extraction table, see S3 and S4 Tables. All data extraction was performed by two authors (MU and TO) independently, followed by consensus meetings in case of differences, using a third author (TF or VD) to resolve conflicts.

### Data summary and synthesis

The retrieved data were compared between the eligible systematic reviews. A summary table was made describing the review study characteristics. For the data extracted from the economic evaluations included in the systematic review, a second summary table was generated (S4 Table), allowing for descriptive figures. For relevant extracted data from the economic evaluations, findings will be presented in diagrammatic or tabular form. For economic evaluations that were included in multiple systematic reviews, the number of times the evaluations were included in different reviews was denoted. For PGx tests that specified the use of a gene panel, if the genes included were not specified, it was categorised as not aligned with a PGx guideline. In case of differences in extracted information regarding evaluations between reviews, TO and MU agreed on the most relevant source. Inconsistencies between the review studies were reported.

### Risk of bias assessment

The quality of eligible systematic reviews was assessed by the Assessing the Methodological Quality of Systematic Reviews-2 (AMSTAR-2) checklist [19]. AMSTAR-2 consists of 16 items, categorised into critical (seven items) and non-critical (nine items) domains. AMSTAR-2 is originally used for systematic reviews of randomised controlled trials and cohort studies. Therefore, the domains regarding meta-analyses and bias (questions 9, 11, 12, and 15) were skipped as they were not relevant to the reviews of economic evaluations. Specifically, the bias relates to the randomisation and blinding of patients, which is not relevant in an economic evaluation, unless they were directly linked to an experimental study design.

The overall quality of systematic review were graded as: high confidence, if the study was compliant with all items of critical and noncritical domains, or if only one noncritical domain was violated; moderate confidence, if the study was compliant with all critical domains but more than one of the non-critical domains were violated; low confidence if one or critically low confidence, if more than one of the critical domains were violated (regardless of non-critical domains). TO and MU independently assessed the quality of each review study, with any discrepancies first being discussed between the two researchers. In the case that a consensus was not reached, a third investigator (TF or VD) was consulted.

## Results

### Results from systematic reviews

#### Overview of the extraction process.

**Fig 1:**
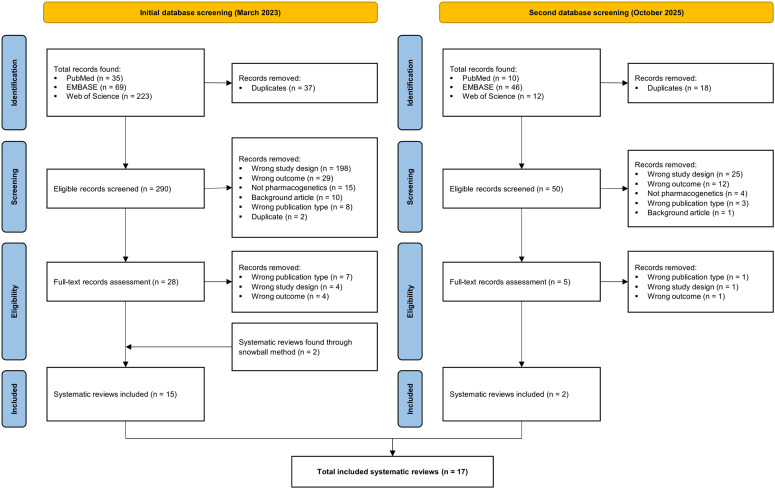
PRISMA flowchart for study selection. A flowchart for the study selection for inclusion in the umbrella review on health economic evaluations of pharmacogenetic‌‌ testing‌‌.

From the initial screening of the three databases, 35 review studies were retrieved from PubMed, 69 studies were retrieved from EMBASE, and 223 studies were retrieved from Web of Science (Fig 1). After applying the initial screening criteria and removal of duplicates, 28 review studies remained for full-text reading. Two additional systematic reviews were found in the study by Berm *et al.*, which fit the eligibility criteria and were included. From the second publication database screening, two additional systematic reviews were identified to fit the eligibility criteria and were added to the data extraction. In the end, 17 systematic reviews were included for the umbrella review [8–10,13,14,20–31].

#### Main findings of systematic reviews.

Of the 17 reviews included, eight studies had a general focus and included pharmacoeconomic evaluations of all PGx studies (Table 2). The remaining nine studies had a specific disease focus, either cardiovascular (n = 6), immunosuppression (n = 2), or psychiatric diseases (n = 1). The reviews discussed between six to 108 economic evaluations of PGx studies. Studies before 2011 did not specify using a specific quality assessment. Two studies assessed quality with CHEC-Extended (Consensus Health Economic Criteria list), two studies assessed quality with CHEERS (Consolidated Health Economic Evaluation Reporting Standards), and six used QHES (Quality of Health Economic Studies) as a quality assessment checklist. Six out of 17 reviews were funded through PGx implementation or related programs (S3 Table).

**Table 2:** Overview of included systematic reviews‌‌.

Author	Date range	# studies	Type of studies	Quality assessment	Overall quality included studies^1^	Main conclusions regarding cost-effectiveness^1^
Compagni et al. (2008) [25]	1995 - 2006	7	Disease-specific (Immunosuppression)	No^a^	Poor (Author raises this as a limitation)	Net cost of performing *TPMT* testing to avert one case of neutropenia was ∼€5,300/averted case in 2008.
Vegter et al. (2008) [26]	2000 - 2007	20	General	No^b^	NR	Application of genetic screening was mostly found to be a cost-effective or cost-saving strategy compared to non-screening strategies.
Payne et al. (2009) [24]	1980 - 2008	6	Disease-specific (Immunosuppression)	No^b^	The quality of reporting was variable.	All studies found that *TPMT* testing was cost-effective.
Beaulieu et al. (2010) [14]	2004 - 2010	15	General	No^a^	NR	Future pharmacoeconomic assessments should include every specific component that has an impact on the cost–effectiveness.
Vegter et al. (2010) [23]	2000 - 2010	42	General	No^a^	NR	Increase in the number and quality of evaluations of PGx and genomic screening programmes. However, there are still gaps in information, and the quality of studies should be improved.
Berm et al. (2016) [12]	2010 - 2014	38	General	QHES	Average score of 76	Application of PGx tests was mostly found to be a cost-effective or cost-saving strategy.
Plothner et al. (2016) [22]	2000 - 2015	27	General	QHES	Average score of 85.81	Test-guided personalised therapies were mostly found to be a cost-effective strategy. compared to non-test-guided personalised therapies. To guarantee the cost-effectiveness comparability of stratified drug therapies, national and international standards for evaluation studies should be defined.
Plumpton et al. (2016) [13]	1980 - 2015	47	General	CHEERS	36 studies reported a scoring >85% (considered high)	Cost-effectiveness of testing was supported for multiple gene-drug pairs. Further analyses and/or availability of robust clinical evidence are necessary to make recommendations for the remaining PGx tests.
AlMukdad S et al. (2020) [21]	2011 - 2019	13	Disease-specific (Cardiovascular disease)	QHES	Good: 79–94	*CYP2C19* genotyped-guided antiplatelet therapy is cost-effective compared to the universal use of antiplatelets in patients with ACS undergoing PCI.
Zhu et al. (2020) [15]	2004-2018	46	Disease-specific (Cardiovascular disease)	QHES	Range: 53–100	Evidence on the cost-effectiveness of the treatment of cardiovascular disease is mixed, though generally supportive for select gene-drug relationships.
Karamperis et al. (2021) [16]	2005 - 2020	18	Disease-specific (Psychiatry)	QHES	Average: 87.2; Range: 73–98	50% of the studies reached a conclusion that PGx testing is cost-effective (N = 6) or could be cost-effective (N = 4).
Lim et al. (2021) [20]	2000 - 2015	10	Disease-specific (Cardiovascular disease)	CHEC-Extended	Only mentions items that were reported by all studies.	It was not possible to pinpoint which type of genetic testing would be cost-effective.
Turongkaravee et al. (2021) [19]	2002-2018	59	General	CHEERS	Most studies complied with the checklist, except for single study-based economic evaluations.	Conclusions given per disease area. Uncertainty analysis should be performed and reported on, as well as a rating system for the quality of evidence.
Kamil et al. (2022) [17]	2009 - 2020	18	Disease-specific (Cardiovascular disease)	CHEC-Extended	The number of items rated yes ranged from 8 to 18.	PGx testing to determine coumadin dosing was cost-effective, with approximately half of the comparisons being cost-effective. None of the studies discussed the ethical and distributional issues of genetic testing.
Morris et al. (2022) [18]	2002 - 2021	108	General	QHES	87% (n = 94) had a high quality (score >75)	About three-quarters of articles determined PGx testing was either cost-effective or cost-saving, based on single-gene tests. There is evidence lacking for some PGx tests.
Lim et al. (2024) [29]	2011 - 2022	35	Disease-specific (Cardiovascular disease)	CHEC-Extended	Average: 76.2%; Range: 30–90	PGx to stratify patients for antiplatelet therapy is unlikely to be cost-effective for ticagrelor as standard care, but may be cost-saving or cost-effective for prasugrel or clopidogrel.
Samprasit et al. (2025) [30]	2000 - 2024	6	Disease-specific (Cardiovascular disease)	ECOBIAS	Not reported	PGx-guided dual antiplatelet therapy with cilostazol, ticagrelor, or clopidogrel can be cost-effective or cost-saving compared to universal clopidogrel.

^1^per the review authors, rephrased for more uniformity by current authors. ^a^ No specific methodology specified. ^b^ ISPOR Guidelines mentioned. NR: Not reported. QHES: Quality of Health Economic Studies.

CHEC: Consensus Health Economic Criteria. CHEERS: Consolidated Health Economic Evaluation Reporting Standards. ACS: acute coronary syndrome. PCI: percutaneous coronary intervention.

The conclusions from the systematic reviews were that the number of evaluations concerning the cost-effectiveness of PGx testing was increasing over the years. However, a lack of a standardised approach to study design, minimal clinical effectiveness data, and the use of observational data, rather than randomised clinical trials, were issues raised consistently by the systematic review authors.

#### Quality assessment of systematic reviews.

The AMSTAR-2 tool assessment of the included studies determined two reviews (Lim et al. 2024, Turongkaravee et al. 2021) to be of high quality (Table 3) [22,30]. Four reviews (Morris et al. 2022, Karamperis et al. 2021, Zhu 2020 et al., Plumpton et al. 2016) scored moderate quality [10,13,14,21]. The remaining studies were either of Low or critically low quality (n = 11).

**Table 3:** Quality assessment of included review studies according to the AMSTAR-2 tool.

	AMSTAR-2																
Author, Year	1	2*	3	4*	5	6	7*	8	9*	10	11*	12	13*	14	15*	16	Final Score
Compagni et al. 2008 [25]	Y	N	Y	Y	N	N	PY	N	N/A	N	N/A	N/A	N	Y	N/A	N	Critically low
Vegter et al. 2008 [26]	Y	PY	Y	Y	Y	N	PY	PY	N/A	N	N/A	N/A	N	Y	N/A	Y	Critically low
Payne et al. 2009 [24]	Y	PY	Y	Y	Y	N	Y	PY	N/A	N	N/A	N/A	N	Y	N/A	Y	Critically low
Beaulieu et al. 2010 [14]	Y	PY	Y	Y	N	N	Y	Y	N/A	N	N/A	N/A	N	Y	N/A	Y	Critically low
Vegter et al. 2010 [23]	Y	PY	Y	N	Y	N	N	PY	N/A	Y	N/A	N/A	N	Y	N/A	Y	Critically low
Berm et al. 2016 [12]	Y	PY	Y	N	N	N	Y	PY	N/A	Y	N/A	N/A	Y	Y	N/A	Y	Low
Plöthner et al. 2016 [22]	Y	PY	Y	Y	Y	N	Y	Y	N/A	Y	N/A	N/A	N	Y	N/A	Y	Low
Plumpton et al. 2016 [13]	Y	Y	Y	Y	Y	N	Y	Y	N/A	N	N/A	N/A	Y	Y	N/A	Y	Moderate
AlMukdad et al. 2020 [21]	Y	Y	Y	Y	Y	Y	N	Y	N/A	Y	N/A	N/A	Y	Y	N/A	Y	Low
Zhu et al. 2020 [15]	Y	PY	Y	Y	Y	Y	PY	Y	N/A	Y	N/A	N/A	Y	Y	N/A	Y	Moderate
Karamperis et al. 2021 [16]	Y	PY	Y	Y	Y	Y	Y	Y	N/A	Y	N/A	N/A	Y	Y	N/A	Y	Moderate
Lim et al. 2021 [20]	Y	Y	Y	Y	Y	Y	Y	Y	N/A	Y	N/A	N/A	N	Y	N/A	Y	Low
Turongkaravee et al. 2021 [19]	Y	Y	Y	Y	Y	Y	Y	Y	N/A	Y	N/A	N/A	Y	Y	N/A	Y	High
Kamil et al. 2022 [17]	Y	Y	Y	Y	Y	Y	Y	Y	N/A	Y	N/A	N/A	N	Y	N/A	Y	Low
Morris et al. 2022 [18]	Y	PY	Y	Y	Y	Y	Y	Y	N/A	Y	N/A	N/A	Y	Y	N/A	Y	Moderate
Lim et al. 2024 [29]	Y	Y	Y	Y	Y	Y	Y	Y	N/A	Y	N/A	N/A	Y	Y	N/A	Y	High
Samprasit et al. 2025 [30]	Y	Y	N	Y	Y	N	N	PY	N/A	N	N/A	N/A	Y	Y	N/A	Y	Low

* denotes critical domains of AMSTAR-2. Y, Yes. PY, Potentially yes. N, No. N/A, Not applicable.

### Results from extracted economic evaluations

#### Study characteristics of extracted studies.

In total, data on the results from 515 economic evaluations were extracted from the 17 systematic reviews. Of these, 106 evaluations appeared in multiple systematic reviews, with two studies being included in up to nine systematic reviews (Priest et al 2006, Winter et al 2006). Combining and deduplicating the extracted data, 211 unique evaluation studies were investigated further. Full breakdown of duplicates can be found in S5 Table.

#### Geographical breakdown.

**Fig 2:**
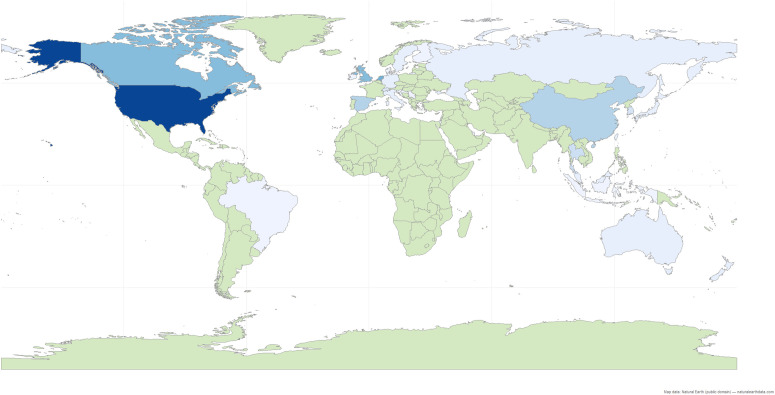
Geographical breakdown of health economic evaluations of (pharmaco)genetic testing. Darker blue denotes a higher number of health economic evaluations. Grey denotes no health economic evaluations. (for full details‌‌ see S3 Table)‌‌.

A total of 31 countries were represented in the evaluations (Fig 2, S6 Table). 105 studies were from the North American continent (United States, n = 92; Canada, n = 13). Europe was the second most represented continent with 62 studies across 16 countries (Top 3 countries represented: The Netherlands, n = 15; United Kingdom, n = 13; Spain, n = 8). Asia was represented by 35 studies across six countries (Top 3 countries represented: Singapore, n = 10; China, n = 8; Thailand, n = 5). South America was represented by one study from Brazil. There were no studies from the African continent.

#### Health economic evaluation results.

**Fig 3:**
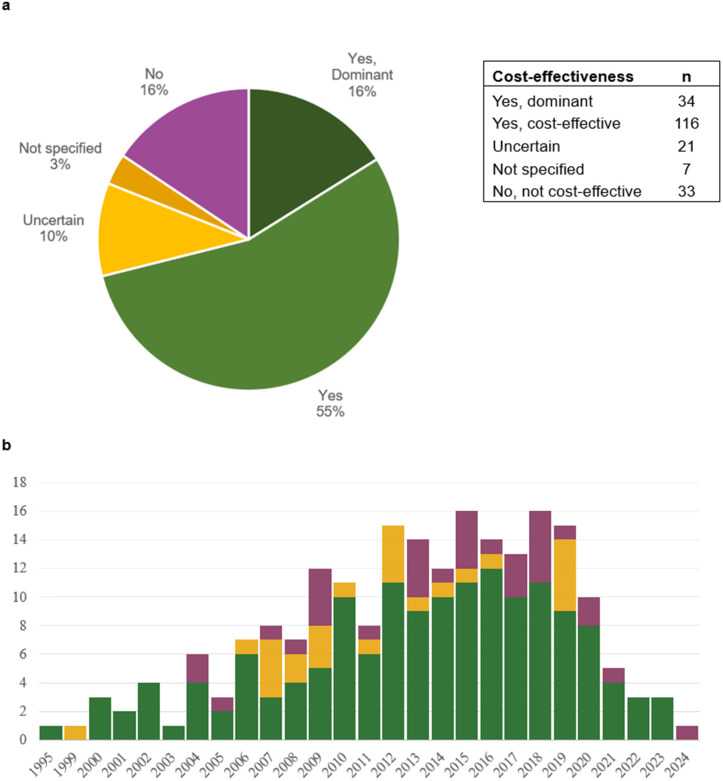
Overview of the conclusions regarding the cost-effectiveness of the included health economic evaluations. **a)** Uncertain denotes that the study reported not being able to assess whether the genetic test was cost-effective or not. Not specified indicates that no information regarding conclusions was found in the information extracted by the selected reviews. **b)** Health economic evaluations of pharmacogenetic tests over time by cost-effectiveness. Green denotes the evaluation determined testing to be cost-effective. Yellow denotes evaluations with uncertain or unspecified cost-effectiveness. Purple denotes the evaluation determined testing to be not cost-effective.

150 out of 211 (71.1%) studies reported in their conclusions that PGx testing was cost-effective (Fig 3a). Within these 150 studies, 34 studies (22.7%) demonstrated that the intervention was dominant (i.e., using PGx to inform drug choice was both improving health outcomes and lowering costs). 33 studies (15.6%) reported finding PGx screening to be not cost-effective. 21 studies (9.9%) found the results to be inconclusive, and seven studies (3.3%) did not specify whether the evaluation was cost-effective. Looking at the trend of cost-effectiveness over time, a steady increase in the number of health economic evaluations from the first study in 1995 can be seen (Fig 3b). In relation to the increasing number of studies, the consistency of economic evaluations demonstrating cost-effectiveness can be seen, sharply increasing from 2010 to 2020. Of the studies that were not-cost-effective during this time window, nearly half (n = 9 of 21) were conducted in Asian countries. Since 2020, the number of economic evaluations included in the systematic reviews decreased, but most evaluations demonstrated cost-effectiveness. Of note, this kind of numbers should be carefully interpreted, since they do not account for study quality and assume that study conclusions were justified.

The most frequently used type of economic evaluation was cost-utility analysis (n = 78) (S7 Table). On the other hand, 45 studies conducted a cost-effectiveness evaluation. Roughly one-third of evaluations did not report the type of economic evaluation used (n = 66). Of the remaining 21 studies, eight were cost-minimisation and cost-analyses, three were a combined cost-effectiveness/cost-utility analysis, and two were cost-benefit analyses. The healthcare perspective was most prominent (n = 75), closely followed by a Payer’s perspective (n = 50) (S7 Table). In total, 32 studies took a Societal perspective, and four studies reported both a Healthcare and Societal perspective. Almost a quarter of the studies did not specify the type of economic perspective (n = 50).

#### Drug classification.

**Fig 4:**
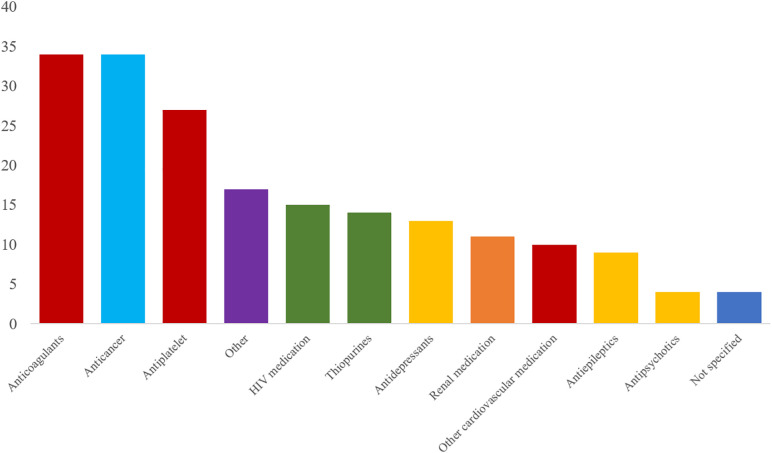
Classification of drugs investigated in the included evaluation studies. The red columns denote cardiovascular drugs. The orange columns denote psychotherapeutic drugs. The green columns denote immunosuppressant drugs. The light blue column denotes all anticancer drugs. The purple column denotes all remaining types of drugs. See S8 Table.

The most evaluated drugs were cardiovascular drugs (n = 85) (Fig 4, S8 Table). Broken down, this consisted of anticoagulants (n = 34, of which 23 were warfarin), antiplatelet drugs (n = 36, of which 21 were clopidogrel) and other cardiovascular drugs (n = 15, of which 4 were statins). Anticancer drugs were investigated in 34 evaluation studies (n = 6 for trastuzumab and fluoropyrimidines). Immunosuppressant drugs (n = 30) were split into drugs for treating HIV (n = 16) and thiopurines (n = 14). Studies evaluating psychiatric drugs (n = 26) could be split into those evaluating antidepressants (n = 13), antiepileptics (n = 9) and antipsychotics (n = 4).

#### Genomic breakdown.

**Fig 5:**
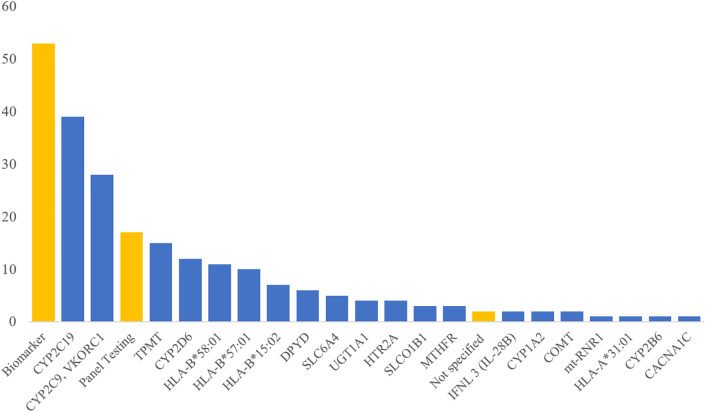
Classification of pharmacogenes investigated in the included evaluation studies. Blue bars denote genes that are included in one of the PGx guidelines (DPWG, CPIC). Yellow bars denote genes that are not included in a PGx guideline. Biomarkers include all genetic biomarkers that were evaluated but not included in the pharmacogenetic guidelines. Breakdown found in S9 Table. Panel Testing refers to studies where a specific pharmacogene was not specified, but a gene panel was used for the evaluation.

Most economic evaluations investigated the pharmacogene *CYP2C19* (n = 46), followed closely by *CYP2C9/VKORC1* (n = 28) (Fig 5, S9 Table). Although the systematic reviews aimed to evaluate PGx testing within the included reviews, 80 evaluations did not correspond to any of the established PGx guidelines. The majority of these evaluations were on genetic biomarkers (n = 53). Of which, six evaluations were conducted for *HER2,* and four evaluations were conducted on *EGFR*. Of the remaining evaluations, 25 evaluations were on gene panels, and two studies did not specify the type of genetic testing that was conducted.

#### Findings from five countries.

Economic evaluations from the five countries (i.e., United States, the Netherlands, Canada, the United Kingdom, and Singapore) comprised 68.7% of the total studies (145 out of 211) (Table 4). Each country had at least one economic evaluation within the top four drug classification categories (i.e., cardiovascular, anticancer, immunosuppressant, psychiatric). Other countries did not have economic evaluations in the four drug classification categories. Economic evaluations of psychiatric drugs had the highest proportion of cost-effectiveness found across the five countries (89%, 17 studies out of 19).

**Table 4:** Breakdown of economic evaluations where cost-effectiveness was concluded when comparing PGx interventions to care as usual by drug classifications in the top five countries.

Country	Drug classification (%, evaluation count)			
	Cardiovascular	Anticancer	Immunosuppressant	Psychiatric
United States	64% (25/39)*	67% (8/12)*	60% (6/10)*	100% (14/14)
The Netherlands	100% (4/4)	100% (2/2)	100% (1/1)	0% (0/2)*
Canada	75% (3/4)	0% (0/2)	75% (3/4)	100% (1/1)
United Kingdom	67% (2/3)*	0% (0/2)*	50% (2/4)*	100% (1/1)
Singapore	100% (2/2)	100% (1/1)	0% (0/2)*	100% (1/1)
Cumulative	69% (36/52)	58% (11/19)	62% (13/21)	89% (17/19)

*The denominator includes economic evaluations that reported uncertain results on cost-effectiveness.

## Discussion

Over the years, health economic evaluations of PGx studies have increased in number and more often concluded that PGx testing was cost-effective. These findings are most relevant to the specific countries in which these studies were performed, and should be interpreted with caution, since study results were not quality assessed. Hence, a study was considered as cost-effective when the study authors made this conclusion. The findings from the second literature search support this statement, as the number of publications determining cost-effectiveness increased in the last two years. The area of application of the PGx tests increased, as well as the range of countries where the evaluations were conducted. For economic evaluations conducted since 2010, the number of studies per year and the respective proportion of cost-effectiveness have increased steadily until 2020. The sharp drop in the number of economic evaluations published and included in reviews since then may be due to the COVID-19 pandemic shifting priorities regarding the conducting of economic evaluations of PGx. In addition, as our study is based on health economic evaluations included in systematic reviews, with only two systematic reviews conducted since 2022, it is likely that our investigation misses economic evaluations published since 2020 and not included in these two systematic reviews.

Considering the top five countries where economic evaluations were conducted, cost-effectiveness was determined for pharmacogenetic testing in psychotherapeutic medications. Economic evaluations of cardiovascular medications were also determined to be cost-effective, albeit a handful of evaluations reported uncertainty due to the high cost of conducting a genetic test. However, it should be kept in mind that many of the studies were evaluated in the United States. Therefore, the transferability of the findings to countries with different healthcare settings, where evaluations have not yet been conducted, remains unclear.

Systematic reviews conducted before 2017 were either focused on evaluations of specific drug-gene interactions (i.e., *TPMT* and azathioprine) [27,28] or were a general review on any (pharmaco)genetic testing [8–10,25,26,29]. On the other hand, more recent systematic reviews focused on disease classifications, which provided more insight into the applicability of PGx testing in the disease of interest [13,14,20,23,24]. This shift in investigation focus may stem from the increased number of economic evaluations of PGx testing. Results of these systematic reviews suggest that PGx testing is cost-effective for patients taking cardiovascular, anticancer, immunosuppressant, or psychiatric drugs. While conclusions were harder to derive from the general systematic reviews for PGx testing, from the systematic reviews that targeted a specific disease, it was identified that barriers for implementation are lowered for the relevant drug-gene interactions if cost-effectiveness can be shown. Furthermore, when gene variants have implications for multiple diseases and comorbidities exist, health benefits and possibly cost savings could be underestimated in evaluations targeting single diseases.

Of note, findings from economic evaluations are strongly setting specific and require careful transferability checking when translated to another location [32]. Additionally this investigation did not quality check all included evaluations, as it is outside the scope of an umbrella review. Hence, the issues raised by reviewers with regard to minimal clinical effectiveness data and use of observational data could not be further explored. Also, the included systematic reviews did not factor in the different cost structures associated with the tests; therefore, comparisons between early studies and later studies were not conducted. For countries where evaluations were not conducted, the current overview provides an opportunity to transfer and work to replicate promising findings adjusted to the local healthcare setting, taking into account local costs of healthcare, variance in the prevalence of certain genetic variants and their own standard of care [33,34].

Nearly 40% of evaluations in the systematic reviews investigated genetic biomarkers that were not listed in the PGx guidelines (i.e., DPWG, CPIC, CPNDS, RNPGx) [1,7,35]. Systematic reviews with a general focus included genetic biomarkers, which comprised pharmacogenes but also other genetic biomarkers that have been associated with treatment response. For earlier systematic reviews with a general focus, the number of evaluations available may have been limited. Therefore, the authors either may not have kept to a strict definition of PGx testing or used a broader definition of genetic testing when conducting their systematic reviews. Additionally, changes in PGx guidelines since the first evaluation were conducted may have influenced this result. Most notably, PGx guidelines regarding *F5* genotypes and the use of contraceptives have changed over time. Within our review, ten evaluations concerned *F5* genotypes, representing roughly 15% of the evaluations not aligned to a PGx guideline. Finally, evaluations that reported using gene panels, but did not specify the genes included, were categorised as outside PGx guidelines. For these evaluations, it could not be ascertained whether a pharmacogene listed in the PGx guidelines was included.

Several challenges to deriving conclusions regarding the general/global cost-effectiveness of PGx testing exist. Different countries use different thresholds to determine whether or not an intervention can be considered cost-effective. For example, studies performed in the United States mentioned a cost-effectiveness threshold of $50,000 to $100.000 per QALY (quality adjusted life years) [36]. Whereas in Canada, thresholds varied per province between CAD $50,000 and CAD $100,000 per QALY [37]. Looking at the European countries, the United Kingdom has a threshold range between £20,000 and £30,000 per QALY [38]. In the Netherlands, the threshold ranges from € 20.000 up to €80.000 per QALY, depending on disease burden [39]. This implies that a study conducted in the US could conclude testing to be cost-effective, while the study results would not lead to this conclusion in the UK, based on the lower threshold applied in the UK, even ignoring any further transferability issues. From the extracted evaluations, the proportion of evaluations that were considered cost-effective in the UK (6 studies out of 12) was lower than this proportion in the US (62 studies out of 87). Therefore, it is not possible to conclude in general that decreasing the cost of genetic testing would lead to PGx testing being cost-effective in all countries.

Another issue concerning the transferability of evaluation results was the gaps found in the data presented. Evaluations that were covered by multiple systematic studies showed worrying differences in extracted data. Furthermore, information regarding the type of economic evaluation was limited, as one-third of studies did not report this parameter. Differences in perspective also impact whether a PGx test can be considered cost-effective, and were not reported in almost a quarter of all studies. In this umbrella review, a distinction was made between the healthcare and payer’s perspective. It was difficult to follow the categorisation extracted from the systematic reviews as there were discrepancies in terminology. This may reflect how different countries view the difference between the healthcare system and payers [33,34,40–42]. One direction to address these gaps is for the alignment of the evaluations to established criteria such as CHEERS, CHEC-Extended or QHES [43–45]. Next to these criteria, for economic evaluations of PGx testing, we recommend the following information to be routinely included to support transferability of results to other settings: the genetic variation of the local population and expanded details on models used, i.e., applying good practice guidelines for decision-analytic next to CHEERS [46].

While gene panels were included in the evaluations, the cost-effectiveness of gene panels remains unclear. This stems from the relatively small number of evaluations which investigated gene panels and the large variation in study settings. As gene panels cover a broad scope of pharmacogenes, the applicability of a selected gene panel for PGx testing in multiple drug-gene interactions would require investigations across different interactions. More recently, the PREPARE-study gene panel, which comprised PGx genes, was economically evaluated [47]. As the cost of gene panel sequencing decreases and the scope of genes covered increases, the relevance to evaluate their cost-utility increases. Compared to single gene tests, the costs of the gene panel may be higher, but the applicability of the data for multiple different diseases would facilitate broader application and more cost-savings. In the future, the genetic data analysed in gene panels may be translated into polygenic risk scores, which may identify the risks of disease as well as drug response by determining the aggregate variations in pharmacokinetic pathways.

The quality of the systematic reviews has improved over the years. This partly aligns with the increasing incorporation of formal quality assessments of the economic evaluations included by the systematic reviews. Specifically, this was seen for the systematic reviews after 2010. From the systematic reviews included, three different quality assessments were used (i.e., QHES, CHEC-Extended, CHEERS). The AMSTAR-2 tool was, however, developed for the evaluation of systematic reviews of healthcare interventions (RCTs and cohort studies) [19]. Economic evaluations are usually not suitable for a meta-analysis due to differences in methods, locations and other variables. Furthermore, none of the reviews performed a quantitative evidence synthesis. This implied that four out of 16 criteria of AMSTAR-2 were not applicable. Therefore, the conclusion regarding the quality of systematic reviews may be less reliable. While several systematic reviews were critical of aspects of study quality, as explained in the results, most economic evaluations that were assessed for quality scored high on study quality.

### Strengths and limitations

The umbrella review offers a historical overview of the developments in economic evaluations of PGx testing. Improvements in the quality of methodology and expansion of evaluations across different countries were seen over the years. It could be seen that more economic evaluations were conducted over time, in different settings, while the costs of genetic testing decreased. However, as the search was limited to including studies available from the selected systematic reviews, publication bias cannot be excluded. Only a handful (n = 5) of studies were conducted by an industrial party promoting their gene panel. Therefore, the publication bias due to commercial interests is probably limited. On the other hand, publication bias from the economic evaluations that concluded cost-effectiveness, but had poor reporting on the type of economic analysis or economic perspective, may be warranted. Although the inclusion criteria were meant to encompass all drug-gene interactions in PGx guidelines, not all have had evaluations conducted. Therefore, whether the cost-effectiveness is representative for all PGx testing needs further study.

As previous systematic reviews focused on limited scopes (i.e., a specific disease) or provided limited information (i.e., missing data), this umbrella review provides a more complete overview of health economic evaluations of PGx testing. Even the most recent general systematic review covered only half the studies that were found in the current umbrella review (108 vs 211 studies), underlining the relevance of an umbrella review [21]. With more than half the included studies comprising a combination of extracted data from multiple studies, the final combination represents a consensus of the extracted data from different systematic reviews, which also could be considered a benefit of an umbrella review. On the other hand, the discrepancy of extracted information between evaluations may be considered a limitation. As the authors did not read each economic evaluation study’s full text, it could not be ascertained which extracted data were correct. Especially, data extracted concerning the time horizon studied and the type of effects included varied frequently. Whether this discrepancy stems from the lack of clarity in the original manuscript or a lack of health economics/pharmacogenetics expertise within the systematic review authors was unclear. It would be recommendable that future systematic reviews specify how the data from the evaluations were extracted. This includes, but is not limited to, detailing data extraction methods and performing double and independent data extraction.

As the investigation was limited to systematic reviews, which in the large majority only searched for evaluations conducted in the English language, a publication bias based on linguistic scope may exist. A quick scan of literature in other languages found some economic evaluations of PGx testing [48–51]. It may be reasoned that a broader systematic review including different languages may shed light on the cost-effectiveness in countries where English is not the main language.

## Conclusions

Over the years, the number of economic evaluations concluding the cost-effectiveness of pharmacogenetic testing has increased proportionally. However, since many of the evaluation findings of this study were from the United States, a like-for-like interpretation of conclusions is not possible. It is important to keep in mind that cost-effectiveness is dependent on the country where the evaluation is conducted and will vary by local thresholds for cost-effectiveness, disease, gene type, study design, time horizon and perspective considered. Given the large number of studies that conclude the cost-effectiveness of PGx testing in cardiovascular and psychotherapeutic drugs, the consolidation of these evaluations would present a strong case for the reimbursement of PGx testing in patients using these medications. Furthermore, future evaluations should also include non-English evaluations. In countries where the cost-effectiveness of PGx testing has not yet been evaluated, the findings of the umbrella review present an opportunity to conduct initial assessments. The transferability to countries where evaluations have not yet been conducted may not be out of reach, as current health economic policy practice indicates that the use of evaluations from other countries is possible [52,53]. However, due to the potential difference in the prevalence of genetic variants in pharmacogenes, considerations for the ethnic groups in a country are required when trying to transfer findings. Looking to the future, as genetic sequencing technology advances, the cost of PGx testing of multiple pharmacogenes (i.e., gene panels) will continue to decrease. This will reduce the cost barrier of PGx testing and lead to greater accessibility for optimised treatment.

## Supporting information

S1 Table PRISMA 2020 Checklist. (DOCX)

S2 Table Search terms for PubMed, EMBASE, and Web of Science Databases. Selection for Reviews/Systematic Reviews was filtered within the database.
(DOCX)

S3 Table Full list of systematic review information. (XLSX)

S4 Table Full list of extracted studies from the systematic reviews. Combined denotes multiple systematic reviews that included the economic evaluation.
(XLSX)

S5 Table Synthesis of combined studies. (XLSX)

S6 Table Breakdown of economic evaluations of pharmacogenetic studies. (XLSX)

S7 Table Overview of economic analysis and perspective. (DOCX)

S8 Table Breakdown of all investigated medications. (XLSX)

S9 Table Breakdown of all investigated genes and biomarkers. (XLSX)
