# Supplementary material for: Cost-effectiveness of pre-emptive pharmacogenetic testing: An umbrella review
Source: PLoS One. 2026 Jun 16;21(6):e0338277. doi: 10.1371/journal.pone.0338277 (PMC13271446; doi:10.1371/journal.pone.0338277)
Supplement: S2 Table — Selection for Reviews/Systematic Reviews was filtered within the database. (DOCX) [file pone.0338277.s002.docx]

**Supplementary Table 2. Search terms for PubMed, EMBASE and Web of Science Databases**

| Database | Search terms |
| --- | --- |
| PubMed | ("Economics, Pharmaceutical"[Mesh] OR “pharmaceutical economic*”[tiab] OR “pharmacoeconomic*”[tiab] OR “pharmaco economic*”[tiab] OR “pharmacy economic*”[tiab] OR "Cost-Benefit Analysis"[Mesh] OR “cost benefit analys*”[tiab] OR “cost and benefit*”[tiab] OR “benefits and cost*”[tiab] OR “cost effectiveness*”[tiab] OR “cost benefit data”[tiab] OR “cost utility analys*”[tiab] OR “economic evaluation*”[tiab] OR “marginal analys*”[tiab] OR “economic impact*”[tiab])  AND  ("Pharmacogenetics"[Mesh] OR “pharmacogenetic*”[tiab] OR “pharmacogenomic*”[tiab] OR “genetic*”[tiab] OR “genotype*”[tiab] OR "Polymorphism, Single Nucleotide"[Mesh] OR “single Nucleotide Polymorphism*”[tiab] OR “SNP”[tiab] OR “SNPs”[tiab] OR "Biomarkers"[Mesh] OR “biomarker*”[tiab] OR “biological marker*”[tiab] OR “biologic marker*”[tiab])  AND  ("Precision Medicine"[Mesh] OR “Drug-Related Side Effects and Adverse Reactions “[Mesh] OR “precision medicine*”[tiab] OR “personalised medicine*”[tiab] OR “personalized medicine*”[tiab] OR “P health*”[tiab] OR “Pgx test*” [tiab] OR “adverse drug reaction*” [tiab]) |
| EMBASE | ('pharmacoeconomics'/exp OR 'pharmacoeconomics' OR 'pharmaceutical economic*':ab,ti OR 'pharmaco economic*':ab,ti OR 'pharmacy economic*':ab,ti OR 'cost effectiveness analysis':ab,ti OR 'cost benefit analysis'/exp OR 'cost benefit analysis' OR 'cost and benefit*':ab,ti OR 'benefits and cost*':ab,ti OR 'cost benefit data':ab,ti OR 'cost utility analysis':ab,ti OR 'economic evaluation':ab,ti OR 'marginal analys*':ab,ti OR 'sensitivity analysis':ab,ti OR 'economic impact*')  AND  ('pharmacogenetics'/exp OR 'pharmacogenetics' OR 'pharmacogenetic*':ab,ti OR 'pharmacogenomic*':ab,ti OR 'genetic*':ab,ti OR 'genotype*':ab,ti OR 'genetic screening':ab,ti OR 'single nucleotide polymorphism'/exp OR 'single nucleotide polymorphism' OR 'snp':ab,ti OR 'snps':ab,ti OR 'biological marker'/exp OR 'biological marker' OR 'biomarker*':ab,ti OR 'biologic marker*':ab,ti)  AND  ('personalized medicine'/exp OR 'personalized medicine' OR 'precision medicine*':ab,ti OR 'pgx test*':ab,ti OR 'p health*':ab,ti OR 'adverse drug reaction*':ab,ti) AND  ([cochrane review]/lim OR [systematic review]/lim OR [meta analysis]/lim) |
| Web of Science | ("Economics, Pharmaceutical" OR “pharmaceutical economic*” OR “pharmacoeconomic*” OR “pharmaco economic*” OR “pharmacy economic*” OR "Cost-Benefit Analysis" OR “cost benefit analys*” OR “cost and benefit*” OR “benefits and cost*” OR “cost effectiveness*” OR “cost benefit data” OR “cost utility analys*” OR “economic evaluation*” OR “marginal analys*” OR “economic impact*”)  AND  ("Pharmacogenetics" OR “pharmacogenetic*” OR “pharmacogenomic*” OR “genetic*” OR “genotype*” OR "Polymorphism, Single Nucleotide" OR “single Nucleotide Polymorphism*” OR “SNP” OR “SNPs” OR "Biomarkers" OR “biomarker*” OR “biological marker*” OR “biologic marker*”)  AND ("Precision Medicine" OR “Drug-Related Side Effects and Adverse Reactions “ OR “precision medicine*" OR “personalised medicine*” OR “personalized medicine*” OR “P health*” OR “Pgx test*” OR “adverse drug reaction*”) |

Selection for Reviews/Systematic Reviews was filtered within in database.
