# Supplementary material for: Cost-effectiveness of pre-emptive pharmacogenetic testing: An umbrella review
Source: PLoS One. 2026 Jun 16;21(6):e0338277. doi: 10.1371/journal.pone.0338277 (PMC13271446; doi:10.1371/journal.pone.0338277)
Supplement: S7 Table — (DOCX) [file pone.0338277.s007.docx]

**_Supplementary Table 7. Overview of economic analysis and perspective_**

_(a) Type of economic analysis_

| **_Type of economic analysis_** | **_N_** |
| --- | --- |
| _Cost-utility analysis (CUA)_ | _78_ |
| _Cost-effectiveness analysis (CEA)_ | _45_ |
| _Cost-minimisation analysis (CMA)_ | _8_ |
| _Cost analysis (CA)_ | _8_ |
| _Cost benefit analysis (CBA)_ | _3_ |
| _CUA/CEA_ | _3_ |
| _Not specified_ | _66_ |

_(b) Type of economic perspective_

| **_Type of economic perspective_** | **_n_** |
| --- | --- |
| _Healthcare_ | _75_ |
| _Payers_ | _50_ |
| _Societal_ | _32_ |
| _Healthcare + Societal_ | _4_ |
| _Not specified_ | _50_ |
