# Supplementary material for: Cost-effectiveness of pre-emptive pharmacogenetic testing: An umbrella review
Source: PLoS One. 2026 Jun 16;21(6):e0338277. doi: 10.1371/journal.pone.0338277 (PMC13271446; doi:10.1371/journal.pone.0338277)
Supplement: Attachment — Submitted filename: Response to reviewers PLOS ONE_mar2026_def.pdf [file pone.0338277.s011.pdf]

UMC Utrecht

30 March 2026

Subject: Response to Reviewers for manuscript "Cost-effectiveness of pre-emptive pharmacogenetic testing: an umbrella review" (Manuscript ID: PONE-D-25-60772)

Dear Editor,

We thank you for your careful evaluation of our manuscript and for the constructive comments on our manuscript. Below, we provide a point-by-point response to each of your comments. We have made revisions to improve the manuscript based on the reviewers' suggestions and hope that with these revisions, the manuscript will be accepted for publication in PLOS ONE.

Editorial comments
Editorial comment 1:
Please ensure that your manuscript meets PLOS ONE's style requirements, including those for file naming. The PLOS ONE style templates can be found at:
https://journals.plos.org/plosone/s/file?id=wjVg/PLOSOne_formatting_sample_main_body.pdf and
https://journals.plos.org/plosone/s/file?id=ba62/PLOSOne_formatting_sample_title_authors_affiliations.pdf

Author response:
The manuscript has been checked, and the needed changes to meet PLOS ONE's style requirements have been made.

Editorial comment 2:
Please amend your authorship list in your manuscript file to include author Manon Germa den Uil.

Author response:
The authorship list in the manuscript was complete; Manon Germa den Uil is the second author in the list (Manon G den Uil). Both Taichi Ochi and Manon G den Uil contributed equally to this manuscript.

Editorial comment 3:
Please amend the manuscript submission data (via Edit Submission) to include author Manon G den Ui.

Author response:
Manon G den Uil is the same author as Manon Germa den Uil.

Editorial comment 4:
We note that Figure 2 in your submission contain [map/satellite] images which may be copyrighted. All PLOS content is published under the Creative Commons Attribution License (CC BY 4.0), which means that the manuscript, images, and Supporting Information files will be freely available online, and any third party is permitted to access, download, copy, distribute, and use these materials in any way, even commercially, with proper attribution. For these reasons, we cannot publish previously copyrighted maps or satellite images created using proprietary data, such as Google software (Google Maps, Street View, and Earth). For more information, see our copyright guidelines: http://journals.plos.org/plosone/s/licenses-and-copyright.

We require you to either (1) present written permission from the copyright holder to publish these figures specifically under the CC BY 4.0 license, or (2) remove the figures from your submission.

Author response:
We appreciate the editorial comment regarding Figure 2. After enquiring for written permission and not receiving a response, the authors used the literature provided regarding the alternative options available to publish a world map. The current Figure 2 uses R package, ‘rnaturalearth’ to which we the added data from our study, ourselves. We have acknowledged the map data in the Figure legend, based on how to use the package Natural Earth. So, no copyright issue is involved that needs to be resolved.

Editorial comment 5:
If the reviewer comments include a recommendation to cite specific previously published works, please review and evaluate these publications to determine whether they are relevant and should be cited. There is no requirement to cite these works unless the editor has indicated otherwise.

Author response:
The reviewer comments did not include a recommendation to cite specific previously published works; no additional citations were added.

Reviewers comments

Reviewer: 1

This umbrella review provides a valuable synthesis of PGx cost-effectiveness evidence, strengthening implementation arguments amid falling test costs. Strengths: Comprehensive search, transparent methods, visualizations, and identification of trends/gaps.

Author's Response:

Dear Reviewer, thank you for your comments. We are happy to hear your positive input and highlighting the strengths of our manuscript so succinctly.

Reviewer: 2

Major comment 1: Add a clear time-stratified view (how has cost-effectiveness changed over time?)
A key driver in PGx economics is that genotyping costs have decreased substantially over time, and clinical implementation has also changed. Right now, the manuscript provides a pooled picture across many years, but it does not clearly show whether cost-effectiveness conclusions are becoming more favourable over time (which is the intuitive expectation).

What I would suggest:
Please add a simple time-stratified summary (e.g., by decade, or pre/post 2010 or 2015), showing how the proportion of "cost-effective" conclusions changes across periods. Even a basic figure would help readers see whether the evidence is shifting in a meaningful way.

This would make the paper more than an evidence catalogue and would improve its usefulness for interpretation.

Author's response:
First of all, we would like to thank the reviewer for taking the time to review the article and providing us with useful information and comments on the manuscript. The feedback was greatly appreciated and helped us to improve our article.

We have made a time-stratified bar chart (Figure 3b) that breaks down the evaluations over time, colour-coding the studies as cost-effective, uncertain, or not cost-effective. In addition, we added a line denoting the percentage of evaluations that were cost-effective with respect to the year. This provides further insight into how cost-effectiveness has changed over time. Additional text has been added to the Results and Discussion to expand on the findings of this graph:

Results, Line 245-253:
Looking at the trend of cost-effectiveness over time, a steady increase in the number of health economic evaluations from the first study in 1995 can be seen (Fig 3b). In relation to the increasing number of studies, the consistency of economic evaluations demonstrating cost-

effectiveness can be seen, sharply increasing from 2010 to 2020. Of the studies that were not-cost-effective during this time window, nearly half (n = 9 of 21) were conducted in Asian countries. Since 2020, the number of economic evaluations included in the systematic reviews decreased, but most evaluations demonstrated cost-effectiveness. Of note, this kind of numbers should be carefully interpreted, since they do not account for study quality and assume that study conclusions were justified.

Results, Line 266-269:
Fig 3. a) [...] b) Health economic evaluations of pharmacogenetic tests over time by cost-effectiveness. Green denotes the evaluation determined testing to be cost-effective. Yellow denotes evaluations with uncertain or unspecified cost-effectiveness. Purple denotes the evaluation determined testing to be not cost-effective.

Discussion, Line 321-327:
For economic evaluations conducted since 2010, the number of studies per year and the respective proportion of cost-effectiveness have increased steadily until 2020. The sharp drop in the number of economic evaluations published and included in reviews since then may be due to the COVID-19 pandemic shifting priorities regarding the conducting of economic evaluations of PGx. In addition, as our study is based on health economic evaluations included in systematic reviews, with only two systematic reviews conducted since 2022, it is likely that our investigation misses economic evaluations published since 2020 and not included in these two systematic reviews.

Major comment 2: Do not treat older and newer evidence as equally informative for “current” conclusions
Related to the above, I think the manuscript currently mixes older evaluations (done under very different test costs and care pathways) with more recent evaluations, and then presents a single headline proportion. For an outcome like cost-effectiveness—where the cost structure evolves with technology—this risks turning the umbrella review into a largely historical overview rather than a robust assessment of whether pre-emptive PGx testing is cost-effective under current conditions.

What I would suggest:
Please provide a clearly labelled “recent evidence” snapshot (e.g., last 5–10 years) and discuss it separately as the most policy-relevant view.

If you keep the full historical synthesis, I would separate it explicitly as a “historical map” and avoid letting it drive strong present-day conclusions. This would also help align the conclusion with what the evidence can reasonably support today.

Author's response:
We greatly appreciate the reviewer’s insightful comments regarding the time-stratified view of cost-effectiveness. We agree that highlighting recent evidence sheds additional information relating to the cost-effectiveness of the studies. Specifically, in the last 10 years, roughly half the studies demonstrating not cost-effective came from Asian countries, representing a third of

evaluations from the region. More details relating to this finding have been added to the Results and Discussion.

Results lines 246-253:
In relation to the increasing number of studies, the consistency of economic evaluations demonstrating cost-effectiveness can be seen, sharply increasing from 2010 to 2020. Of the studies that were not-cost-effective during this time window, nearly half (n = 9 of 21) were conducted in Asian countries. Since 2020, the number of economic evaluations included in the systematic reviews decreased, but most evaluations demonstrated cost-effectiveness. Of note, this kind of numbers should be carefully interpreted, since they do not account for study quality and assume that study conclusions were justified.

Discussion, lines 321-322:
For economic evaluations conducted since 2010, the number of studies per year and the respective proportion of cost-effectiveness have increased steadily until 2020.

Major comment 3: Geographic limitation and selection bias: strengthen the message that the evidence is not truly “global.”

The paper presents a global synthesis, but in practice, the evidence base is heavily concentrated in a small number of countries. The manuscript also notes clear gaps (e.g., no systematic reviews from Africa). This creates two problems:

Generalizability: cost-effectiveness depends on local thresholds, unit costs, standard of care, and population characteristics, so results from one country may not apply elsewhere.

Selection bias in the evidence base: countries that have enough activity to produce systematic reviews may already be settings where PGx implementation is feasible and supported, which can make the overall evidence look more positive than it would be in settings with no published reviews.

What I would suggest:
Please tighten the abstract/discussion/conclusion so the overall “X% cost-effective” message is not read as globally applicable. I would avoid broad statements implying the economic barrier is “minimal” in general. Instead, frame the conclusion as setting- and context-dependent, and emphasise evidence gaps where reviews do not exist.

Author’s response:
We agree that it is important to carefully explain the overall cost-effectiveness, which should not be read as globally applicable. Therefore, we tightened the manuscript where needed. The discussion and conclusion in the original version of the manuscript already addressed results as setting- and context-dependent, and emphasised evidence gaps within the strengths and limitations section, but we stressed this more now throughout the manuscript. The changes, as well as specific places where context-dependent aspects are mentioned, are repeated below.

Abstract's conclusions, lines 52-55 (revision):
The number of economic evaluations demonstrating cost-effectiveness of pharmacogenetic testing has increased over the years. However, the majority of these evaluations were conducted in a small number of countries. When interpreting findings for different countries, the evaluations need to be adjusted on a case-by-case basis for applicability.

Discussion, lines 314-318 (revision):
Over the years, health economic evaluations of PGx studies have increased in number and more often concluded that PGx testing was cost-effective. These findings are most relevant to the specific countries in which these studies were performed, and should be interpreted with caution, since study results were not quality assessed. Hence a study was considered as cost-effective when the study authors made this conclusion.

As well as lines 331-334:
However, it should be kept in mind that many of the studies were evaluated in the United States. Therefore, the transferability of the findings to countries with different healthcare settings, where evaluations have not yet been conducted, remains unclear.

Also, lines 348-357 (revision):
Of note, findings from economic evaluations are strongly setting specific and require careful transferability checking when translated to another location [32]. Additionally, this investigation did not quality check all included evaluations, as it is outside the scope of an umbrella review. Hence, the issues raised by reviewers with regard to minimal clinical effectiveness data and use of observational data could not be further explored. Also, the included systematic reviews did not factor in the different cost structures associated with the tests; therefore, comparisons between early studies and later studies were not conducted. For countries where evaluations were not conducted, the current overview provides an opportunity to transfer and work to replicate promising findings adjusted to the local healthcare setting, taking into account local costs of healthcare, variance in the prevalence of certain genetic variants and their own standard of care [33,34].

Discussion, lines 371-384 (revision):
Several challenges to deriving conclusions regarding the general/global cost-effectiveness of PGx testing exist. Different countries use different thresholds to determine whether or not an intervention can be considered cost-effective. [...] Therefore, it is not possible to conclude in general that decreasing the cost of genetic testing would lead to PGx testing being cost-effective in all countries.

Conclusion, lines 459-463:
However, since many of the evaluation findings of this study were from the United States, a like-for-like interpretation of conclusions is not possible. It is important to keep in mind that cost-effectiveness is dependent on the country where the evaluation is conducted and will vary by local thresholds for cost-effectiveness, disease, gene type, study design, time horizon, and perspective considered.

As well as lines 468-473:
The transferability to countries where evaluations have not yet been conducted may not be out of reach, as current health economic policy practice indicates that the use of evaluations from other countries is possible [50,51]. However, due to the potential difference in the prevalence of genetic variants in pharmacogenes, considerations for the ethnic groups in a country are required when trying to transfer findings.

Minor comment 1: COI/context and small editorial fixes
This is a field where many authors may also be involved in PGx implementation or related programs. I am not suggesting any specific bias in this manuscript, but it would be helpful for interpretation if the paper briefly notes whether included systematic reviews reported COI/funding statements (even a short sentence or small summary is fine).

Also, please correct minor typographical/grammatical issues throughout.

Author's response:
We agree that it is important to note whether included papers reported COI funding statements. All systematic reviews were checked, and no specific COI and/or funding statements were made. The funding statements have been added to the supplementary table 3 (please find an excerpt of this table attached at the end of the letter). Additionally, it is addressed in the manuscript in lines 198-199:

Six out of 17 reviews were funded through PGx implementation or related programs (Table S3).

Lastly, the full manuscript has been checked for minor typographical/grammatical issues throughout and adjusted accordingly.

Thank you again for your time and consideration.

Please address all correspondence regarding this manuscript to m.g.denuil-2@umcutrecht.nl.

Yours sincerely,
On behalf of all authors,

Manon den Uil
PhD candidate | Pharmacist
Division Laboratories, Pharmacy, and Biomedical Genetics | Department Clinical Pharmacy
University Medical Center Utrecht
m.g.denuil-2@umcutrecht.nl | +31 6 31 11 94 28

UMC Utrecht

Attachment

S3 Table. Excerpt from table showing funding and COI statements for included systematic reviews.

Study	Author	Year	Funding/COI (if declared)
Pharmacogenetic-guided antiplatelet therapy for personalized medicine in patients with stroke and transient ischemic attack without cardioembolic stroke: A systematic review of economic view of economic evaluations	Samprasit	2025	The study was supported by the Graduate School, Burapha University through the doctoral study, produced as part of the Pharmaceutical Sciences and Management Graduate Program at the Faculty of Pharmaceutical Sciences, Burapha University in Thailand.
Genetic-Guided Pharmacotherapy for Coronary Artery Disease: A Systematic and Critical Review of Economic Evaluations	Lim	2024	Salary funding was received from the UK National Institute for Health Research-Biomedical Research Centre based at Guy's and St Thomas' National Health Service Foundation Trust and King's College London (K.K.L., J.F.R., and C.D.A.W.), the British Heart Foundation (P.C.) and the UK National Institute for Health and Care Research Programme Grant for Applied Research NIHR202339 (J.F.R. and C.D.A.W.). The views expressed are those of the authors and not necessarily those of the National Health Service, the National Institute for Health and Care Research, the British Heart Foundation, or the Department of Health and Social Care.
Genetic-Guided Pharmacotherapy for Atrial fibrillation: A Systematic and Critical Review of Economic Evaluations.	Kamil	2022	This research was supported by the National Institute for Health Research Biomedical Research Centre based at Guy's and St Thomas' NHS Foundation Trust and King's College London. The views expressed are those of the authors and not necessarily those of the National Health Service, the National Institute for Health Research, or the Department of Health.<br><br>Dr Lim and Ms Fox-Rushby reported receiving payments to their institutions from the Biomedical Research Center. No other disclosures were reported.

Cost Effectiveness of Pharmacogenetic Testing for Drugs with Clinical Pharmacogenetics Implementation Consortium (CPIC) Guidelines: A Systematic Review	Morris	2022	NA
Economic evaluation in psychiatric pharmacogenomics: a systematic review	Karamperis	2021	This study has been partly funded by the European Union’s Horizon 2020 research and innovation grant (H2020’ 668353; Ubiquitous Pharmacogenomics) to GPP and CM and, partly funded by the European Union’s Horizon 2020 research and innovation under the Marie Skłodowska - Curie grant agreement (H2020’ 860895; TranSYS) to GPP.
Pharmacogenetic testing of adverse drug reaction prevention: systematic review of economic evaluations and the appraisal of quality matters for clinical practice and implementation.	Turongkaravee	2021	This work is a part of a study in the Doctor of Philosophy program in Social, Economic and Administrative Pharmacy (SEAP), Faculty of Pharmacy, Mahidol University, Bangkok. Besides, this study was supported by grants from the International Research Network-the Thailand Research Fund (IRN60W003) and Health Systems Research Institute (HSRI61–080), Thailand. However, the funders had no role in the design, conduct of the study; collection, management, analysis and interpretation of data, and preparation of the manuscript.
Genetic-guided pharmacotherapy for venoous thromboembolism: a systematic and critical review of economic evaluations.	Lim	2021	The research was funded by the National Institute for Health Research (NIHR) Biomedical Research Centre based at Guy’s and St Thomas’ NHS Foundation Trust and King’s College London. The views expressed are those of the authors and not necessarily those of the NHS, the NIHR or the Department of Health.
Economic Evaluations of CYP2C19 Genotype-Guided Antiplatelet Therapy compared to the Universal Use of Antiplatelets in Patients With Acute Coronary Syndrome: A Systematic Review	AlMukdad S, Elewa H, Al-Badriyeh D	2020	The author(s) disclosed receipt of the following financial support for the research, authorship, and/or publication of this article: This research is supported by GSRA award (GSRA5-2-0521-18060) from Qatar National Research Fund and local student Qatar University Grant (QUST-1-CPH-2019-16).
Systematic review of the evidence on the cost-effectiveness of pharmacogenomics-guided treatment for cardiovascular diseases	Zhu	2020	This research was partially supported by a research grant from Mayo Clinic Robert D. and Patricia E. Kern Center for the Science of Health Care Delivery and Mayo Clinic Center for Individualized Medicine. The authors affirm that this paper is an honest, accurate, and transparent account of

			the study being reported; that no important aspects of the study have been omitted; and that any discrepancies from the study as planned have been explained. The views expressed in the article are those of the authors and do not necessarily reflect the views of Mayo Clinic.<br><br>None of the authors have any potential conflicts of interest with respect to the authorship and/or publication of this article. None of the authors reported any financial relationships with any organizations that might have an interest in the submitted work in the previous three years. RW and LW are co-founders of and stock-holders in OneOme LLC, a pharmacogenomic decision support company. There are no other relationships or activities that could appear to have influenced the submitted work.
Cost-Effectiveness of Pharmacogenomic and Pharmacogenetic Test-Guided Personalized Therapies: A Systematic Review of the Approved Active Substances for Personalized Medicine in Germany	Plothner	2016	NA
Economic Evaluations of Pharmacogenetic and Pharmacogenomic Screening Tests: ASystematic Review. Second Update of the Literature	Berm	2016	The funder provided support in the form of salaries for authors CB, SV, but did not have any additional role in the study design, data collection and analysis, decision to publish, or preparation of the manuscript. The specific roles of these authors are articulated in the 'author contributions' section. No funding was received for this study. GSK provided support in the form of salaries for authors CB, SV, but did not have any additional role in the study design, data collection and analysis, decision to publish, or preparation of the manuscript. The specific roles of these authors are articulated in the 'author contributions' section. Maarten J. Postma and Lieven Annemans have received grants, honoraria and speaking fees from several pharmaceutical companies. No other potential conflict of interest related to this study was reported.<br><br>The authors have the following interests: Stefan Vegter and Cornelis Boersma are employees of GSK, besides their part-time academic

			positions. Their contribution to this manuscript was from their visiting researcher positions they hold at the University of Groningen. Maarten J. Postma and Lieven Annemans have received grants, honoraria and speaking fees from several pharmaceutical companies (Abbott Diagnostics, Bayer, Biomarin, Boehringer, Bresmed, Forest labs, Genzyme, Gmasol, GSK, GSK bio, Ingress Health, Mundipharma, Novartis, PerkinElmer, Pfizer, Pharmerit, Roche, Sandoz, Sanofi, Shire, SPMSD, Vertex). No other potential conflict of interest related to this study was reported. There are no patents, products in development or marketed products to declare.
A Systematic Review of Economic Evaluations of Pharmacogenetic Testing for Prevention of Adverse Drug Reactions	Plumpton	2016	This report is independent research funded by the National Institute for Health Research Invention for Innovation Programme (A biomarker panel to predict, diagnose and prevent HLA-mediated ADRs; reference number II-LB-0313-20008).
Systematic review of pharmacoeconomic studies of pharmacogenomic tests	Beaulieu	2010	NA
Economic Evaluations of Pharmacogenetic and Genomic Screening Programs: Update of the Literature	Vegter	2010	This work was supported by the Health Investments in Screening and Diagnosis (HIScreenDiag) project, funded by the European Commission under the 7th Framework Programme Theme: Health, FP7-Health-2007-B, 3.4.2 Effectiveness of Health Investments (project 223533), for the HIScreenDiag project. Parts of this work were supported by the applied GENomic strategies for Treatment and Prevention of Cardiovascular death in Uraemia and End stage REnal disease (GENECURE) project (www.genecure.eu), funded by the European Commission under the 6th Framework Programme as FP6-037696.
TPMT testing in azathioprine: a 'cost-effective use of healthcare resources'?	Payne	2009	This paper is based on the 2007 European Commission JRC Report ‘Improving pharmacovigilance in Europe: Cost–effectiveness analysis of CYP2D6 and TPMT Genotyping in general clinical practice‘. Funded by the European Commission.
Avoiding adverse drug reactions by pharmacogenetic testing: A systematic	Compagni	2008	This work was carried out under contract with the European Commission, Institute of Prospective Technological Studies (IPTS), contract no. 150083-2005-02 BE.

review of the economic evidence in the case of TPMT and AZA-induced side effects			
Pharmacoeconomic Evaluations of Pharmacogenetic and Genomic Screening Programmes: A Systematic Review on Content and Adherence to Guidelines	Vegter	2008	This work was supported by the applied GENomic stratEgies for Treatment and Prevention of Cardiovascular death in Uraemia and End stage REnal disease (GENECURE) project (www.genecure.eu), a Specific Targeted Research or Innovation Project, funded by the European Commission under the Sixth Framework Programme as FP6−037696. GENECURE is led by Professor Dr G.J. Navis, University Medical Center Groningen in Groningen, the Netherlands; its goal is to elucidate the genomic basis of cardiovascular complications in renal disease. GENECURE is hosted by the Renal Genome Network (ReGeNet) project (www.regenet.eu), a pan-European network of clinicians and scientists from academia and industry seeking to generate and facilitate genetic and genomic studies to the clinical benefit of the renal patient.<br><br>This article has been prepared with the assistance of the European Union. The content of this review is the sole responsibility of the authors and can in no way be taken to reflect the views of the European Union. The authors have no conflicts of interest that are directly relevant to the content of this review.
