# Supplementary material for: Cost-effectiveness of pre-emptive pharmacogenetic testing: An umbrella review
Source: PLoS One. 2026 Jun 16;21(6):e0338277. doi: 10.1371/journal.pone.0338277 (PMC13271446; doi:10.1371/journal.pone.0338277)
Supplement: Attachment — Submitted filename: Rebuttal letter PLOS ONE_nov2025.docx [file pone.0338277.s010.docx]

11 November 2025

Subject: Rebuttal letter for manuscript “Cost-effectiveness of pre-emptive pharmacogenetic testing: an umbrella review” (Original manuscript ID: PONE-D-25-48204)

Dear Editor,

We thank you for your careful evaluation of our manuscript and the constructive comments that have helped us to improve the quality and clarity of our work. We have substantially revised the manuscript in response to the concerns raised. Below, we provide a detailed point-by-point response to each comment, indicating where changes have been made in the revised manuscript.

We hope that the revisions satisfactorily address all the issues raised, and we look forward to your reconsideration of our work for publication in PLOS ONE.

Editorial comment 1:

*“In the case of your submission, we note that language restrictions have been imposed on the literature searches without adequate justification. There is increasing recognition that the exclusion of studies published in other languages can increase the risk of bias in systematic reviews. We feel that any restrictions should be justified based on scientific reasons rather than resource constraints. As a result, we have concerns about the comprehensiveness of the review and the validity of the conclusions.”*

Response:

We have revised the literature search to remove language restrictions. This update did not result in the inclusion to any additional articles. See page 5, lines 71-77 of the revised manuscript.

Editorial comment 2:

*“In addition, we are concerned that the literature review was completed over a year ago. Systematic and scoping reviews should provide an up-to-date overview of the research topic covered, and we do not feel that this study meets this requirement.”*

Response:

We have updated our search to include studies published up to October 2025, ensuring the review provides an up-to-date and comprehensive overview of the topic. This update resulted in the inclusion of two additional articles. See page 4, lines 52-54, and page 7, lines 127-129 of the revised manuscript. Corresponding updates have been made throughout the Results and Discussion sections. All revised changes are highlighted in the version with tracked changes.

Additional revision:

Following the PLOS ONE author guidelines, we have also revised the manuscript to align more closely with the PRISMA guidelines for systematic reviews, where applicable for our umbrella review. These revisions ensure a clearly defined research question and a transparent, reproducible methodology. See page 4, lines 48-49, and Supplementary Table 1.

Thank you again for your time and consideration.

Please address all correspondence regarding this manuscript to me at [m.g.denuil-2@umcutrecht.nl](mailto:m.g.denuil-2@umcutrecht.nl)

Yours sincerely,

On behalf of all authors,

Manon den Uil

PhD candidate | Pharmacist

Division Laboratories, Pharmacy, and Biomedical Genetics | Department Clinical Pharmacy

University Medical Center Utrecht

[m.g.denuil-2@umcutrecht.nl](mailto:m.g.denuil-2@umcutrecht.nl) | +31 6 31 11 94 28
