# Supplementary material for: Cost-effectiveness of pre-emptive pharmacogenetic testing: An umbrella review
Source: PLoS One. 2026 Jun 16;21(6):e0338277. doi: 10.1371/journal.pone.0338277 (PMC13271446; doi:10.1371/journal.pone.0338277)
Supplement: Peer review history

# Author comment: Author response to Decision Letter 0

**DOI:** 10.1371/journal.pone.0338277.r001

21 Nov 2025

Attachment Submitted filename: Rebuttal letter PLOS ONE_nov2025.docx

# Peer review: Decision Letter 0

**DOI:** 10.1371/journal.pone.0338277.r002

**Authors:** Nejat Mahdieh

15 Feb 2026

PONE-D-25-60772

Cost-effectiveness of pre-emptive pharmacogenetic testing: an umbrella review

PLOS One

Dear Dr. den Uil,

Thank you for submitting your manuscript to PLOS ONE. After careful consideration, we feel that it has merit but does not fully meet PLOS ONE’s publication criteria as it currently stands. Therefore, we invite you to submit a revised version of the manuscript that addresses the points raised during the review process.

Please submit your revised manuscript by Apr 01 2026 11:59PM. If you will need more time than this to complete your revisions, please reply to this message or contact the journal office at plosone@plos.org. When you're ready to submit your revision, log on to https://www.editorialmanager.com/pone/ and select the 'Submissions Needing Revision' folder to locate your manuscript file.

Please include the following items when submitting your revised manuscript:

- A letter that responds to each point raised by the academic editor and reviewer(s). You should upload this letter as a separate file labeled 'Response to Reviewers'.
- A marked-up copy of your manuscript that highlights changes made to the original version. You should upload this as a separate file labeled 'Revised Manuscript with Track Changes'.
- An unmarked version of your revised paper without tracked changes. You should upload this as a separate file labeled 'Manuscript'.

If you would like to make changes to your financial disclosure, please include your updated statement in your cover letter. Guidelines for resubmitting your figure files are available below the reviewer comments at the end of this letter.

If applicable, we recommend that you deposit your laboratory protocols in protocols.io to enhance the reproducibility of your results. Protocols.io assigns your protocol its own identifier (DOI) so that it can be cited independently in the future. For instructions see: https://journals.plos.org/plosone/s/submission-guidelines#loc-laboratory-protocols. Additionally, PLOS ONE offers an option for publishing peer-reviewed Lab Protocol articles, which describe protocols hosted on protocols.io. Read more information on sharing protocols at https://plos.org/protocols?utm_medium=editorial-email&utm_source=authorletters&utm_campaign=protocols.

We look forward to receiving your revised manuscript.

Kind regards,

Nejat Mahdieh

Academic Editor

PLOS One

Journal Requirements:

When submitting your revision, we need you to address these additional requirements.

1. Please ensure that your manuscript meets PLOS ONE's style requirements, including those for file naming. The PLOS ONE style templates can be found at

https://journals.plos.org/plosone/s/file?id=wjVg/PLOSOne_formatting_sample_main_body.pdf and https://journals.plos.org/plosone/s/file?id=ba62/PLOSOne_formatting_sample_title_authors_affiliations.pdf

2. Please amend your authorship list in your manuscript file to include author Manon Germa den Uil.

3. Please amend the manuscript submission data (via Edit Submission) to include author Manon G den Ui.

4. We note that Figure 2 in your submission contain [map/satellite] images which may be copyrighted. All PLOS content is published under the Creative Commons Attribution License (CC BY 4.0), which means that the manuscript, images, and Supporting Information files will be freely available online, and any third party is permitted to access, download, copy, distribute, and use these materials in any way, even commercially, with proper attribution. For these reasons, we cannot publish previously copyrighted maps or satellite images created using proprietary data, such as Google software (Google Maps, Street View, and Earth). For more information, see our copyright guidelines: http://journals.plos.org/plosone/s/licenses-and-copyright.

We require you to either (1) present written permission from the copyright holder to publish these figures specifically under the CC BY 4.0 license, or (2) remove the figures from your submission:

a. You may seek permission from the original copyright holder of Figure 2 to publish the content specifically under the CC BY 4.0 license.

We recommend that you contact the original copyright holder with the Content Permission Form (http://journals.plos.org/plosone/s/file?id=7c09/content-permission-form.pdf) and the following text:

“I request permission for the open-access journal PLOS ONE to publish XXX under the Creative Commons Attribution License (CCAL) CC BY 4.0 (http://creativecommons.org/licenses/by/4.0/). Please be aware that this license allows unrestricted use and distribution, even commercially, by third parties. Please reply and provide explicit written permission to publish XXX under a CC BY license and complete the attached form.”

Please upload the completed Content Permission Form or other proof of granted permissions as an "Other" file with your submission.

In the figure caption of the copyrighted figure, please include the following text: “Reprinted from [ref] under a CC BY license, with permission from [name of publisher], original copyright [original copyright year].”

b. If you are unable to obtain permission from the original copyright holder to publish these figures under the CC BY 4.0 license or if the copyright holder’s requirements are incompatible with the CC BY 4.0 license, please either i) remove the figure or ii) supply a replacement figure that complies with the CC BY 4.0 license. Please check copyright information on all replacement figures and update the figure caption with source information. If applicable, please specify in the figure caption text when a figure is similar but not identical to the original image and is therefore for illustrative purposes only.

The following resources for replacing copyrighted map figures may be helpful:

USGS National Map Viewer (public domain): http://viewer.nationalmap.gov/viewer/

The Gateway to Astronaut Photography of Earth (public domain): http://eol.jsc.nasa.gov/sseop/clickmap/

Maps at the CIA (public domain): https://www.cia.gov/library/publications/the-world-factbook/index.html and https://www.cia.gov/library/publications/cia-maps-publications/index.html

NASA Earth Observatory (public domain): http://earthobservatory.nasa.gov/

Landsat: http://landsat.visibleearth.nasa.gov/

USGS EROS (Earth Resources Observatory and Science (EROS) Center) (public domain): http://eros.usgs.gov/#

Natural Earth (public domain): http://www.naturalearthdata.com/

5. If the reviewer comments include a recommendation to cite specific previously published works, please review and evaluate these publications to determine whether they are relevant and should be cited. There is no requirement to cite these works unless the editor has indicated otherwise.

[Note: HTML markup is below. Please do not edit.]

Reviewers' comments:

Reviewer's Responses to Questions

**Comments to the Author**

1. Is the manuscript technically sound, and do the data support the conclusions?

The manuscript must describe a technically sound piece of scientific research with data that supports the conclusions. Experiments must have been conducted rigorously, with appropriate controls, replication, and sample sizes. The conclusions must be drawn appropriately based on the data presented.

Reviewer #1: Yes

Reviewer #2: Partly

**********

2. Has the statistical analysis been performed appropriately and rigorously?

Reviewer #1: Yes

Reviewer #2: Yes

**********

3. Have the authors made all data underlying the findings in their manuscript fully available?

The PLOS Data policy requires authors to make all data underlying the findings described in their manuscript fully available without restriction, with rare exception (please refer to the Data Availability Statement in the manuscript PDF file). The data should be provided as part of the manuscript or its supporting information, or deposited to a public repository. For example, in addition to summary statistics, the data points behind means, medians and variance measures should be available. If there are restrictions on publicly sharing data—e.g. participant privacy or use of data from a third party—those must be specified.

Reviewer #1: Yes

Reviewer #2: Yes

**********

4. Is the manuscript presented in an intelligible fashion and written in standard English?

PLOS ONE does not copyedit accepted manuscripts, so the language in submitted articles must be clear, correct, and unambiguous. Any typographical or grammatical errors should be corrected at revision, so please note any specific errors here.

Reviewer #1: Yes

Reviewer #2: Yes

**********

5. Review Comments to the Author

Please use the space provided to explain your answers to the questions above. You may also include additional comments for the author, including concerns about dual publication, research ethics, or publication ethics. (Please upload your review as an attachment if it exceeds 20,000 characters)

Reviewer #1: This umbrella review provides a valuable synthesis of PGx cost-effectiveness evidence, strengthening implementation arguments amid falling test costs. Strengths: Comprehensive search, transparent methods, visualizations, and identification of trends/gaps.

Reviewer #2: <overall summary=“”>

This is a timely topic and an umbrella review can be useful because the PGx cost-effectiveness literature is scattered across many evaluations and multiple systematic reviews. That said, I struggled with the “so what, for today?” interpretation. In PGx, costs and implementation conditions have changed dramatically over time, and economic results do not transfer cleanly across countries. These issues need to be more central to the synthesis and conclusions.

*Major comment 1 — Add a clear time-stratified view (how has cost-effectiveness changed over time?)

A key driver in PGx economics is that genotyping costs have decreased substantially over time, and clinical implementation has also changed. Right now, the manuscript provides a pooled picture across many years, but it does not clearly show whether cost-effectiveness conclusions are becoming more favorable over time (which is the intuitive expectation).

What I would suggest:

Please add a simple time-stratified summary (e.g., by decade, or pre/post 2010 or 2015), showing how the proportion of “cost-effective” conclusions changes across periods.

Even a basic figure would help readers see whether the evidence is shifting in a meaningful way.

This would make the paper more than an evidence catalogue and would improve its usefulness for interpretation.

*Major comment 2 — Do not treat older and newer evidence as equally informative for “current” conclusions

Related to the above, I think the manuscript currently mixes older evaluations (done under very different test costs and care pathways) with more recent evaluations, and then presents a single headline proportion. For an outcome like cost-effectiveness—where the cost structure evolves with technology—this risks turning the umbrella review into a largely historical overview rather than a robust assessment of whether pre-emptive PGx testing is cost-effective under current conditions.

What I would suggest:

Please provide a clearly labeled “recent evidence” snapshot (e.g., last 5–10 years) and discuss it separately as the most policy-relevant view.

If you keep the full historical synthesis, I would separate it explicitly as a “historical map” and avoid letting it drive strong present-day conclusions.

This would also help align the conclusion with what the evidence can reasonably support today.

*Major comment 3 — Geographic limitation and selection bias: strengthen the message that the evidence is not truly “global”

The paper presents a global synthesis, but in practice the evidence base is heavily concentrated in a small number of countries. The manuscript also notes clear gaps (e.g., no systematic reviews from Africa). This creates two problems:

Generalizability: cost-effectiveness depends on local thresholds, unit costs, standard of care, and population characteristics, so results from one country may not apply elsewhere.

Selection bias in the evidence base: countries that have enough activity to produce systematic reviews may already be settings where PGx implementation is feasible and supported, which can make the overall evidence look more positive than it would be in settings with no published reviews.

What I would suggest:

Please tighten the abstract/discussion/conclusion so the overall “X% cost-effective” message is not read as globally applicable. I would avoid broad statements implying the economic barrier is “minimal” in general. Instead, frame the conclusion as setting- and context-dependent, and emphasize evidence gaps where reviews do not exist.

*Minor comment 1 — COI/context and small editorial fixes

This is a field where many authors may also be involved in PGx implementation or related programs. I am not suggesting any specific bias in this manuscript, but it would be helpful for interpretation if the paper briefly notes whether included systematic reviews reported COI/funding statements (even a short sentence or small summary is fine).

Also, please correct minor typographical/grammatical issues throughout.</overall>

**********

6. PLOS authors have the option to publish the peer review history of their article (what does this mean?). If published, this will include your full peer review and any attached files.

If you choose “no”, your identity will remain anonymous but your review may still be made public.

**Do you want your identity to be public for this peer review?**   For information about this choice, including consent withdrawal, please see our Privacy Policy.

Reviewer #1: No

Reviewer #2: No

**********

[NOTE: If reviewer comments were submitted as an attachment file, they will be attached to this email and accessible via the submission site. Please log into your account, locate the manuscript record, and check for the action link "View Attachments". If this link does not appear, there are no attachment files.]

To ensure your figures meet our technical requirements, please review our figure guidelines: https://journals.plos.org/plosone/s/figures

You may also use PLOS’s free figure tool, NAAS, to help you prepare publication quality figures: https://journals.plos.org/plosone/s/figures#loc-tools-for-figure-preparation.

NAAS will assess whether your figures meet our technical requirements by comparing each figure against our figure specifications.

# Author comment: Author response to Decision Letter 1

**DOI:** 10.1371/journal.pone.0338277.r003

9 Apr 2026

Letter below has previously been attached as file labled 'Response to Reviewers'.

Dear Editor,

We thank you for your careful evaluation of our manuscript and for the constructive comments on our manuscript. Below, we provide a point-by-point response to each of your comments. We have made revisions to improve the manuscript based on the reviewers' suggestions and hope that with these revisions, the manuscript will be accepted for publication in PLOS ONE.

Editorial comments

Editorial comment 1:

Please ensure that your manuscript meets PLOS ONE's style requirements, including those for file naming. The PLOS ONE style templates can be found at:

https://journals.plos.org/plosone/s/file?id=wjVg/PLOSOne_formatting_sample_main_body.pdf and https://journals.plos.org/plosone/s/file?id=ba62/PLOSOne_formatting_sample_title_authors_affiliations.pdf

Author response:

The manuscript has been checked, and the needed changes to meet PLOS ONE’s style requirements have been made.

Editorial comment 2:

Please amend your authorship list in your manuscript file to include author Manon Germa den Uil.

Author response:

The authorship list in the manuscript was complete; Manon Germa den Uil is the second author in the list (Manon G den Uil). Both Taichi Ochi and Manon G den Uil contributed equally to this manuscript.

Editorial comment 3:

Please amend the manuscript submission data (via Edit Submission) to include author Manon G den Ui.

Author response:

Manon G den Uil is the same author as Manon Germa den Uil.

Editorial comment 4:

We note that Figure 2 in your submission contain [map/satellite] images which may be copyrighted. All PLOS content is published under the Creative Commons Attribution License (CC BY 4.0), which means that the manuscript, images, and Supporting Information files will be freely available online, and any third party is permitted to access, download, copy, distribute, and use these materials in any way, even commercially, with proper attribution. For these reasons, we cannot publish previously copyrighted maps or satellite images created using proprietary data, such as Google software (Google Maps, Street View, and Earth). For more information, see our copyright guidelines: http://journals.plos.org/plosone/s/licenses-and-copyright.

We require you to either (1) present written permission from the copyright holder to publish these figures specifically under the CC BY 4.0 license, or (2) remove the figures from your submission.

Author response:

We appreciate the editorial comment regarding Figure 2. After enquiring for written permission and not receiving a response, the authors used the literature provided regarding the alternative options available to publish a world map. The current Figure 2 uses R package, ‘rnaturalearth’ to which we the added data from our study, ourselves. We have acknowledged the map data in the Figure legend, based on how to use the package Natural Earth. So, no copyright issue is involved that needs to be resolved.

Editorial comment 5:

If the reviewer comments include a recommendation to cite specific previously published works, please review and evaluate these publications to determine whether they are relevant and should be cited. There is no requirement to cite these works unless the editor has indicated otherwise.

Author response:

The reviewer comments did not include a recommendation to cite specific previously published works; no additional citations were added.

Reviewers comments

Reviewer: 1

This umbrella review provides a valuable synthesis of PGx cost-effectiveness evidence, strengthening implementation arguments amid falling test costs. Strengths: Comprehensive search, transparent methods, visualizations, and identification of trends/gaps.

Author’s Response:

Dear Reviewer, thank you for your comments. We are happy to hear your positive input and highlighting the strengths of our manuscript so succinctly.

Reviewer: 2

Major comment 1: Add a clear time-stratified view (how has cost-effectiveness changed over time?)

A key driver in PGx economics is that genotyping costs have decreased substantially over time, and clinical implementation has also changed. Right now, the manuscript provides a pooled picture across many years, but it does not clearly show whether cost-effectiveness conclusions are becoming more favourable over time (which is the intuitive expectation).

What I would suggest:

Please add a simple time-stratified summary (e.g., by decade, or pre/post 2010 or 2015), showing how the proportion of “cost-effective” conclusions changes across periods.

Even a basic figure would help readers see whether the evidence is shifting in a meaningful way.

This would make the paper more than an evidence catalogue and would improve its usefulness for interpretation.

Author’s response:

First of all, we would like to thank the reviewer for taking the time to review the article and providing us with useful information and comments on the manuscript. The feedback was greatly appreciated and helped us to improve our article.

We have made a time-stratified bar chart (Figure 3b) that breaks down the evaluations over time, colour-coding the studies as cost-effective, uncertain, or not cost-effective. In addition, we added a line denoting the percentage of evaluations that were cost-effective with respect to the year. This provides further insight into how cost-effectiveness has changed over time. Additional text has been added to the Results and Discussion to expand on the findings of this graph:

Results, Line 245-253:

Looking at the trend of cost-effectiveness over time, a steady increase in the number of health economic evaluations from the first study in 1995 can be seen (Fig 3b). In relation to the increasing number of studies, the consistency of economic evaluations demonstrating cost-effectiveness can be seen, sharply increasing from 2010 to 2020. Of the studies that were not-cost-effective during this time window, nearly half (n = 9 of 21) were conducted in Asian countries. Since 2020, the number of economic evaluations included in the systematic reviews decreased, but most evaluations demonstrated cost-effectiveness. Of note, this kind of numbers should be carefully interpreted, since they do not account for study quality and assume that study conclusions were justified.

Results, Line 266-269:

Fig 3. a) […] b) Health economic evaluations of pharmacogenetic tests over time by cost-effectiveness. Green denotes the evaluation determined testing to be cost-effective. Yellow denotes evaluations with uncertain or unspecified cost-effectiveness. Purple denotes the evaluation determined testing to be not cost-effective.

Discussion, Line 321-327:

For economic evaluations conducted since 2010, the number of studies per year and the respective proportion of cost-effectiveness have increased steadily until 2020. The sharp drop in the number of economic evaluations published and included in reviews since then may be due to the COVID-19 pandemic shifting priorities regarding the conducting of economic evaluations of PGx. In addition, as our study is based on health economic evaluations included in systematic reviews, with only two systematic reviews conducted since 2022, it is likely that our investigation misses economic evaluations published since 2020 and not included in these two systematic reviews.

Major comment 2: Do not treat older and newer evidence as equally informative for “current” conclusions

Related to the above, I think the manuscript currently mixes older evaluations (done under very different test costs and care pathways) with more recent evaluations, and then presents a single headline proportion. For an outcome like cost-effectiveness—where the cost structure evolves with technology—this risks turning the umbrella review into a largely historical overview rather than a robust assessment of whether pre-emptive PGx testing is cost-effective under current conditions.

What I would suggest:

Please provide a clearly labelled “recent evidence” snapshot (e.g., last 5–10 years) and discuss it separately as the most policy-relevant view.

If you keep the full historical synthesis, I would separate it explicitly as a “historical map” and avoid letting it drive strong present-day conclusions. This would also help align the conclusion with what the evidence can reasonably support today.

Author's response:

We greatly appreciate the reviewer’s insightful comments regarding the time-stratified view of cost-effectiveness. We agree that highlighting recent evidence sheds additional information relating to the cost-effectiveness of the studies. Specifically, in the last 10 years, roughly half the studies demonstrating not cost-effective came from Asian countries, representing a third of evaluations from the region. More details relating to this finding have been added to the Results and Discussion.

Results lines 246-253:

In relation to the increasing number of studies, the consistency of economic evaluations demonstrating cost-effectiveness can be seen, sharply increasing from 2010 to 2020. Of the studies that were not-cost-effective during this time window, nearly half (n = 9 of 21) were conducted in Asian countries. Since 2020, the number of economic evaluations included in the systematic reviews decreased, but most evaluations demonstrated cost-effectiveness. Of note, this kind of numbers should be carefully interpreted, since they do not account for study quality and assume that study conclusions were justified.

Discussion, lines 321-322:

For economic evaluations conducted since 2010, the number of studies per year and the respective proportion of cost-effectiveness have increased steadily until 2020.

Major comment 3: Geographic limitation and selection bias: strengthen the message that the evidence is not truly “global.”

The paper presents a global synthesis, but in practice, the evidence base is heavily concentrated in a small number of countries. The manuscript also notes clear gaps (e.g., no systematic reviews from Africa). This creates two problems:

Generalizability: cost-effectiveness depends on local thresholds, unit costs, standard of care, and population characteristics, so results from one country may not apply elsewhere.

Selection bias in the evidence base: countries that have enough activity to produce systematic reviews may already be settings where PGx implementation is feasible and supported, which can make the overall evidence look more positive than it would be in settings with no published reviews.

What I would suggest:

Please tighten the abstract/discussion/conclusion so the overall “X% cost-effective” message is not read as globally applicable. I would avoid broad statements implying the economic barrier is “minimal” in general. Instead, frame the conclusion as setting- and context-dependent, and emphasise evidence gaps where reviews do not exist.

Author’s response:

We agree that it is important to carefully explain the overall cost-effectiveness, which should not be read as globally applicable. Therefore, we tightened the manuscript where needed. The discussion and conclusion in the original version of the manuscript already addressed results as setting- and context-dependent, and emphasised evidence gaps within the strengths and limitations section, but we stressed this more now throughout the manuscript. The changes, as well as specific places where context-dependent aspects are mentioned, are repeated below.

Abstract’s conclusions, lines 52-55 (revision):

The number of economic evaluations demonstrating cost-effectiveness of pharmacogenetic testing has increased over the years. However, the majority of these evaluations were conducted in a small number of countries. When interpreting findings for different countries, the evaluations need to be adjusted on a case-by-case basis for applicability.

Discussion, lines 314-318 (revision):

Over the years, health economic evaluations of PGx studies have increased in number and more often concluded that PGx testing was cost-effective. These findings are most relevant to the specific countries in which these studies were performed, and should be interpreted with caution, since study results were not quality assessed. Hence a study was considered as cost-effective when the study authors made this conclusion.

As well as lines 331-334:

However, it should be kept in mind that many of the studies were evaluated in the United States. Therefore, the transferability of the findings to countries with different healthcare settings, where evaluations have not yet been conducted, remains unclear.

Also, lines 348-357 (revision):

Of note, findings from economic evaluations are strongly setting specific and require careful transferability checking when translated to another location [32]. Additionally, this investigation did not quality check all included evaluations, as it is outside the scope of an umbrella review. Hence, the issues raised by reviewers with regard to minimal clinical effectiveness data and use of observational data could not be further explored. Also, the included systematic reviews did not factor in the different cost structures associated with the tests; therefore, comparisons between early studies and later studies were not conducted. For countries where evaluations were not conducted, the current overview provides an opportunity to transfer and work to replicate promising findings adjusted to the local healthcare setting, taking into account local costs of healthcare, variance in the prevalence of certain genetic variants and their own standard of care [33,34].

Discussion, lines 371-384 (revision):

Several challenges to deriving conclusions regarding the general/global cost-effectiveness of PGx testing exist. Different countries use different thresholds to determine whether or not an intervention can be considered cost-effective. […] Therefore, it is not possible to conclude in general that decreasing the cost of genetic testing would lead to PGx testing being cost-effective in all countries.

Conclusion, lines 459-463:

However, since many of the evaluation findings of this study were from the United States, a like-for-like interpretation of conclusions is not possible. It is important to keep in mind that cost-effectiveness is dependent on the country where the evaluation is conducted and will vary by local thresholds for cost-effectiveness, disease, gene type, study design, time horizon, and perspective considered.

As well as lines 468-473:

The transferability to countries where evaluations have not yet been conducted may not be out of reach, as current health economic policy practice indicates that the use of evaluations from other countries is possible [50,51]. However, due to the potential difference in the prevalence of genetic variants in pharmacogenes, considerations for the ethnic groups in a country are required when trying to transfer findings.

Minor comment 1: COI/context and small editorial fixes

This is a field where many authors may also be involved in PGx implementation or related programs. I am not suggesting any specific bias in this manuscript, but it would be helpful for interpretation if the paper briefly notes whether included systematic reviews reported COI/funding statements (even a short sentence or small summary is fine).

Also, please correct minor typographical/grammatical issues throughout.

Author’s response:

We agree that it is important to note whether included papers reported COI funding statements. All systematic reviews were checked, and no specific COI and/or funding statements were made. The funding statements have been added to the supplementary table 3 (please find an excerpt of this table attached at the end of the letter). Additionally, it is addressed in the manuscript in lines 198-199:

Six out of 17 reviews were funded through PGx implementation or related programs (Table S3).

Lastly, the full manuscript has been checked for minor typographical/grammatical issues throughout and adjusted accordingly.

Thank you again for your time and consideration.

Yours sincerely,

On behalf of all authors,

M

Attachment Submitted filename: Response to reviewers PLOS ONE_mar2026_def.pdf

# Editor report: Decision Letter 1

**DOI:** 10.1371/journal.pone.0338277.r004

**Authors:** Nejat Mahdieh

2 Jun 2026

Cost-effectiveness of pre-emptive pharmacogenetic testing: an umbrella review

PONE-D-25-60772R1

Dear Dr. Manon Germa den Uil,

We’re pleased to inform you that your manuscript has been judged scientifically suitable for publication and will be formally accepted for publication once it meets all outstanding technical requirements.

Within one week, you’ll receive an e-mail detailing the required amendments. When these have been addressed, you’ll receive a formal acceptance letter and your manuscript will be scheduled for publication.

An invoice will be generated when your article is formally accepted. Please note, if your institution has a publishing partnership with PLOS and your article meets the relevant criteria, all or part of your publication costs will be covered. Please make sure your user information is up-to-date by logging into Editorial Manager at Editorial Manager® and clicking the ‘Update My Information' link at the top of the page. For questions related to billing, please contact billing support.

If your institution or institutions have a press office, please notify them about your upcoming paper to help maximize its impact. If they’ll be preparing press materials, please inform our press team as soon as possible -- no later than 48 hours after receiving the formal acceptance. Your manuscript will remain under strict press embargo until 2 pm Eastern Time on the date of publication. For more information, please contact onepress@plos.org.

Kind regards,

Nejat Mahdieh

Academic Editor

PLOS One

Additional Editor Comments (optional):

Reviewers' comments:

# Editor report: Acceptance letter

**DOI:** 10.1371/journal.pone.0338277.r005

**Authors:** Nejat Mahdieh

PONE-D-25-60772R1

PLOS One

Dear Dr. den Uil,

I'm pleased to inform you that your manuscript has been deemed suitable for publication in PLOS One. Congratulations! Your manuscript is now being handed over to our production team.

At this stage, our production department will prepare your paper for publication. This includes ensuring the following:

* All references, tables, and figures are properly cited

* All relevant supporting information is included in the manuscript submission,

* There are no issues that prevent the paper from being properly typeset

You will receive further instructions from the production team, including instructions on how to review your proof when it is ready. Please keep in mind that we are working through a large volume of accepted articles, so please give us a few days to review your paper and let you know the next and final steps.

Lastly, if your institution or institutions have a press office, please let them know about your upcoming paper now to help maximize its impact. If they'll be preparing press materials, please inform our press team within the next 48 hours. Your manuscript will remain under strict press embargo until 2 pm Eastern Time on the date of publication. For more information, please contact onepress@plos.org.

You will receive an invoice from PLOS for your publication fee after your manuscript has reached the completed accept phase. If you receive an email requesting payment before acceptance or for any other service, this may be a phishing scheme. Learn how to identify phishing emails and protect your accounts at https://explore.plos.org/phishing.

If we can help with anything else, please email us at customercare@plos.org.

Thank you for submitting your work to PLOS ONE and supporting open access.

Kind regards,

PLOS ONE Editorial Office Staff

on behalf of

Dr. Nejat Mahdieh

Academic Editor

PLOS One
